# Multimodal Imaging Reveals Rapid Catecholamine Uptake and Release by Neutrophils

**DOI:** 10.1002/advs.202524193

**Published:** 2026-06-18

**Authors:** Jennifer Mohr, Anne Schmitz, Meshkat Dinarvand, Franziska Wulfert, Sangeetha Shankar, Bjoern F. Hill, Michael Wojak, Juliana Gretz, Marie Britz, Elsa Neubert, Magdalena Shumanska, Sofia Kaushik, Linda Kartaschew, Ivan Bogeski, James Daniel, Sebastian Jung, Johannes Eble, Guido Wabnitz, Luise Erpenbeck, Sebastian Kruss

**Affiliations:** ^1^ Department of Chemistry and Biochemistry Ruhr University Bochum Bochum Germany; ^2^ Department of Dermatology University Hospital Münster Münster Germany; ^3^ Institute of Physical Chemistry Göttingen University Göttingen Germany; ^4^ Department of Dermatology, Venereology and Allergology University Medical Center Göttingen University Göttingen Germany; ^5^ Molecular Physiology, Institute of Cardiovascular Physiology University Medical Center Georg‐August‐University Göttingen Germany; ^6^ Department of Molecular Neurobiology Max Planck Institute For Multidisciplinary Sciences Göttingen Germany; ^7^ ZEMOS Center for Solvation Science Ruhr University Bochum Bochum Germany; ^8^ Institute of Physiological Chemistry and Pathobiochemistry University of Münster Münster Germany; ^9^ Institute For Immunology Heidelberg University Hospital Heidelberg Heidelberg Germany; ^10^ Biomedical Nanosensors Fraunhofer Institute for Microelectronic Circuits and Systems Duisburg Germany

**Keywords:** carbon nanotubes, catecholamines, fluorescence, near‐infrared, neutrophils

## Abstract

Neutrophils are key inflammatory effector cells and rapidly integrate chemical signals during inflammation. They have long been suspected to use catecholamines (CAs) as immunoregulatory signals, but direct evidence for CA handling in these cells has been lacking due to the absence of suitable real‐time detection tools. Here, we combine fluorescent false neurotransmitters (FFNs), near‐infrared fluorescent single‐walled carbon nanotube (SWCNT)‐based catecholamine nanosensors, dual‐color Ca^2^^+^ imaging, and transcriptomics to resolve neutrophil CA dynamics with high spatiotemporal precision. Using FFNs, we demonstrate VMAT2‐dependent vesicular uptake of catecholamines within seconds as well as CA transfer between cells. Serotonin, LPS, and activated platelets trigger calcium (Ca^2+^) signaling and subsequent fast transient release of CAs from neutrophils, which we directly visualize in real time using SWCNT‐based nanosensors. In human experimental endotoxemia, longitudinal transcriptomics reveal coordinated regulation of monoaminergic receptors, synthesis machinery, and transporters, suggesting adaptive tuning of neutrophils to inflammatory CA exposure. CAs suppress NET formation but enhance thrombin‐induced platelet aggregation, and serotonin‐dependent platelet–neutrophil interactions evoke CA release, establishing a paracrine feedback loop linking inflammation and coagulation. Our integrated imaging and sensing workflow provides direct evidence for rapid vesicular catecholamine communication in human neutrophils and uncovers previously unrecognized mechanistic parallels between neurons and neutrophils. It offers a broadly applicable platform to interrogate monoamine signaling in immune cells.

## Graphical abstract

We show that immune cells (neutrophils) synthesize, uptake, and store catecholamine neurotransmitters such as dopamine or adrenaline. They also release them in response to specific stimuli (serotonin), which we directly visualize using fluorescent nanosensors. We further demonstrate that catecholamines affect neutrophil functions (NETosis) and platelet aggregation, which points to a regulatory role of catecholamines in inflammatory and thrombotic disorders.

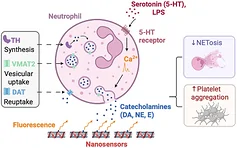

## 1 Introduction

Neutrophilic granulocytes (neutrophils) are key effector cells of the innate immune system and first responders to bacterial and fungal infections. They possess several highly effective defense mechanisms, including phagocytosis, release of antimicrobial substances and reactive oxygen species (ROS), and the formation of neutrophil extracellular traps (NETs) [1, 2]. Dysregulated or excessive neutrophil activation and NETosis contribute to a wide range of inflammatory and malignant diseases and can promote acute tissue damage as well as thromboembolic complications [1, 3, 4]. While NET formation can be influenced by interactions with activated platelets, neutrophils themselves must tightly regulate their activation dynamics, both systemically and during transient local cell–cell encounters. Communication via rapidly diffusing small molecules is well‐suited for such fast and spatially restricted regulation, yet remains incompletely understood.

Catecholamine (CA) neurotransmitters such as dopamine (DA), epinephrine (E), and norepinephrine (NE) are well established as neuromodulators in the central and peripheral nervous system [5]. During exocytosis, secretory vesicles fuse with the cell membrane, which releases the vesicular content into the extracellular space [6]. In immune cells such as neutrophils, exocytosis has been shown to be associated with the release of antimicrobial substances and enzymes to combat infections [7, 8]. However, accumulating evidence indicates a broader role of catecholamines in the immune system. For example, follicular T helper cells release DA to modulate B‐cell activation [9]. Additionally, macrophages, mast cells, and lymphocytes either produce DA or possess DA receptors [10, 11, 12, 13] and adrenergic signaling has been described in granulocytes, macrophages, dendritic cells, natural killer cells, and lymphocytes [14, 15]. For neutrophils, the expression of adrenergic receptors has previously been documented, and anti‐inflammatory effects of ß‐adrenergic effects induced by CA have been established [16, 17, 18, 19, 20]. Yet, these studies have mainly relied on exogenously applied CAs, leaving open the fundamental question of whether neutrophils themselves synthesize, take up, package, and release CAs in a manner analogous to neuronal cells.

Addressing this question has been technically challenging. Conventional biochemical methods such as electrochemical detection, mass spectrometry (MS), or high‐pressure liquid chromatography (HPLC) can detect CAs but lack spatial resolution and, except for electrochemical approaches, sufficient temporal resolution to capture rapid, localized release events [21]. Genetically encoded neurotransmitter sensors provide real‐time readouts but require transfection, which is not feasible in short‐lived primary human neutrophils [22]. These limitations have precluded direct observation of transient, sub‐second catecholamine dynamics in immune cells. Optical nanosensor‐based approaches offer a way to overcome these constraints and have shown potential to image rapid biochemical signaling [23]. Fluorescent single‐walled carbon nanotubes (SWCNTs) operate in the near‐infrared tissue transparency window [24] and can be chemically tailored to detect specific biomolecules, including neurotransmitters [25], lipids, ROS [26] or proteins [27], down to the single molecule level. Such nanosensors have enabled visualization of CA release from pheochromocytoma (PC‐12) cells [28] and primary dopaminergic neurons [29], and other nanosensor designs have been used to image serotonin (5‐HT) exocytosis from activated platelets [30]. In addition, fluorescent false neurotransmitters (FFNs) allow visualization of vesicular monoamine uptake and trafficking via the transporters DAT (dopamine transporter) and VMAT2 (vesicular monoamine transporter 2) in the neuronal system [31].

Here, we combine these complementary tools to directly investigate catecholamine handling in human neutrophils. We demonstrate that neutrophils possess key components of a vesicular monoaminergic machinery and use FFNs to visualize rapid CA uptake and transfer between cells. We further employ SWCNT‐based nanosensors and Ca^2^^+^ imaging to resolve transient CA exocytosis triggered by inflammatory stimuli. Transcriptomic profiling in human experimental endotoxemia reveals dynamic modulation of monoaminergic signaling pathways, supporting an adaptive response to inflammatory catecholamine exposure. Together, our optical platform provides the first real‐time evidence for vesicular catecholamine communication in neutrophils and uncovers previously unrecognized aspects of rapid neutrophil signaling.

## 2 Results

### 2.1 Neutrophils Synthesize, Store, Degrade, and Release Catecholamines

**FIGURE 1:**
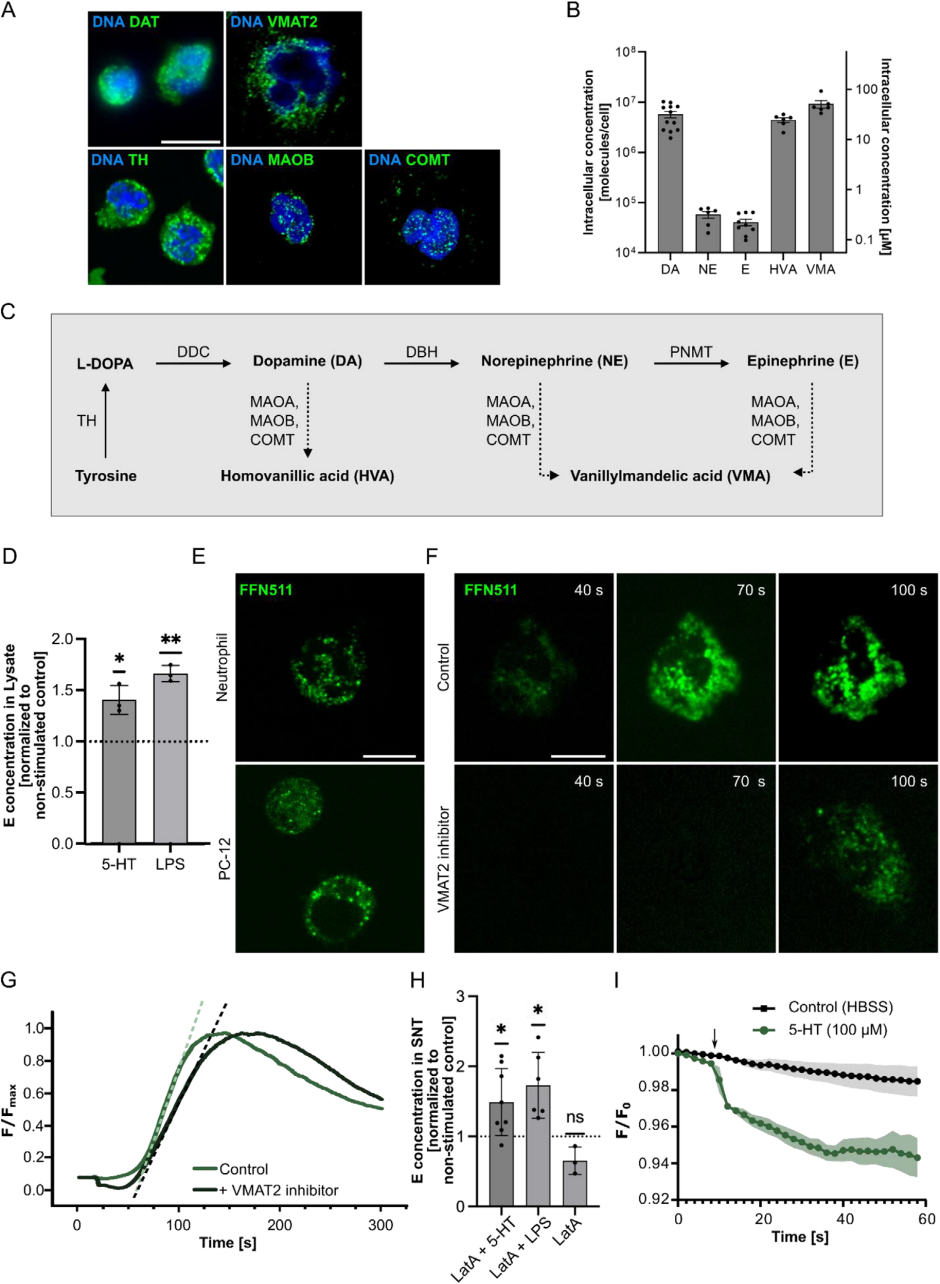
Human neutrophils have the machinery to synthesize, degrade, take up, package, and release catecholamines. (A) Neutrophils are stained for dopamine transporter (DAT), vesicular monoamine transporter 2 (VMAT2), tyrosine hydroxylase (TH), monoamine oxidase B (MAOB), catechol‐O‐methyltransferase (COMT) (all green), and DNA (blue). (Each immunofluorescence staining *n* > 5 donors) Scale bar is 10 µm. (B) Intracellular content of CAs and the metabolic products homovanillic acid (HVA) and vanillylmandelic acid (VMA) determined by an ELISA. (DA: *n* = 12, NE: *n* = 6, E: *n* = 9 donors, mean ± SEM). Assumed neutrophil volume = 10 µm^3^. (C) Scheme of catecholamine metabolism (DDC: DOPA decarboxylase, DBH: dopamine beta‐hydroxylase, PNMT: phenylethanolamine N‐methyltransferase). (D) Epinephrine (E) concentration in lysate after neutrophils were stimulated with 5‐HT (100 µm) or LPS (25 µg/mL) for 30 min, as determined by ELISA (*n* = 3, mean ± SD, one sample *t*‐test: *p*‐value * < 0.05, ** < 0.01). Normalized to the control without stimulation. (E) Image of fluorescent false neurotransmitters (FFN511)‐filled vesicles in live neutrophils and PC‐12 cells (positive control). Scale bar is 10 µm. (F) FFN511 (2.5 nm) uptake of neutrophils with VMAT2 inhibition by tetrabenazine at different time points (G) Corresponding single‐cell kinetic analysis (fluorescence intensity inside the cell) of FFN551 uptake with VMAT2 inhibitor (black) or without (green) and linear fits (dashed lines) (H) Epinephrine (E) concentration in neutrophil supernatant (SNT), normalized to unstimulated samples, after neutrophils had either only been pre‐incubated with latrunculin A (LatA;1 µm)  for 30 min or afterward stimulated either with 5‐HT (100 µm) or LPS (25 µg/mL) for 5 min. Determined by ELISA (*n* = 8 (5‐HT), *n* = 6 (LPS), *n* = 3 (LatA only), mean ± SD, one sample *t*‐test: *p*‐value * < 0.05). (I) FFN511 fluorescence intensity inside cells decreases after stimulation with 5‐HT (100 µm). Note that the decrease in the control trace is due to bleaching (*n* = 3 donors, mean ± SEM (shaded)).

To study whether neutrophils possess the catecholaminergic machinery as described in neuronal cells, we first tested for the presence of VMAT2, DAT, and tyrosine hydroxylase (TH) by immunofluorescence staining (Figure 1A, isotype controls in Figure S2). DAT transports CAs into neurons and VMAT2 is known to package CAs into vesicles [32, 33]. Both are expressed in primary dopaminergic (mouse) neurons (Figure S1) and are essential for neuronal functions. TH is the rate‐limiting enzyme in CA synthesis (standard marker for dopaminergic neurons) and was detected in neutrophils (Figure 1A). Further immunofluorescence staining (Figure 1A and Figure S2) as well as PCR (Figure S3) revealed CA processing and degrading enzymes (monoamine oxidase A, MAOA, and monoamine oxidase B, MAOB) and catechol‐O‐methyltransferase (COMT).

Next, we wanted to assess the presence of the most common catecholamines dopamine (DA), epinephrine (E), or norepinephrine (NE), and, given the presence of degrading enzymes (Figure 1A and overview in 1C), their degradation products. Therefore, an ELISA assay was performed to measure the intracellular concentration of CAs and their metabolites homovanillic acid (HVA) and vanillylmandelic acid (VMA) (Figure 1B). E, NE, and DA as well as the above‐mentioned metabolites were found in neutrophils in different concentrations. While ELISA‐based quantification of small molecules such as catecholamines can be influenced by potential cross‐reactivity and matrix effects, the comparative differences observed between analytes provide reliable relative information on their intracellular abundance. For reference, the number of roughly 10^7^ DA molecules per cell suggests that approximately 300 vesicles could be filled if one assumes a DA content (quantal size) of 33 000 molecules per vesicle. This number matches (in the order of magnitude) the number of vesicles observed in FFN stainings (Figure 1E,F) [34].

Additionally, we aimed to investigate whether inflammatory stimulation alters intracellular CA levels in neutrophils and measured intracellular CA content after *ex vivo* incubation of neutrophils with serotonin (5‐HT) or bacterial lipopolysaccharide (LPS) for 30 min (Figure 1D). Platelet‐derived 5‐HT has been reported to modulate several neutrophil activities, including neutrophil recruitment and degranulation [35], making it an interesting substance to study in the context of CA trafficking, while LPS is generally known to be a potent activator of different neutrophil functions, as warding off bacterial infections is one of the key functions of neutrophils [36]. Indeed, we observed increased CA production after 30 min of incubation with either substance (Figure 1D), which suggests active CA production under inflammatory/pro‐coagulant conditions and is in line with the biphasic upregulation of CA synthesis we later observed in a transcriptomic data set (Figure 6). Of note, these ex vivo experiments were performed in catecholamine‐free media, making reuptake an unlikely explanation for the observed increase in intracellular catecholamine levels and supporting catecholamine synthesis by neutrophils.

In order to directly visualize CA trafficking, we next employed imaging of different fluorescent false neurotransmitters (FFNs) [29]. Neutrophils were first incubated with FFN511, which has a high affinity for VMAT2 [31]. FFN511 was rapidly taken up and then localized in granular structures inside neutrophils (Figure 1E), which is morphologically comparable to the vesicles in the adrenergic PC‐12 cell line (positive control in Figure 1E). To further verify the function of VMAT2, the uptake of FFN511 into neutrophils was observed both under normal conditions and in the presence of the VMAT2 inhibitor tetrabenazine (Figure 1F) [37]. Unspecific uptake of FFNs has been previously reported in cell lines without DAT/VMAT2 and was corroborated in HEK and HeLa cells (Figure S4) [31]. Nevertheless, uptake kinetics of neutrophils under VMAT2 inhibition were significantly slower compared to the controls (Figure 1G), thereby supporting the hypothesis that CA uptake and packaging in neutrophils share functional similarities with neuronal vesicular mechanisms and occur within tens of seconds.

We then wondered what would provoke CA release from neutrophils and tested the above‐mentioned substances 5‐HT and LPS in this context. We employed pre‐incubation with the actin microfilament disruptor latrunculin A for 30 min before the addition of the specific stimulus, as this has been reported to increase degranulation [38, 39], and then added the specific stimulus for 5 min. Neutrophil supernatants were then tested for E content as a representative for all three CA. Both substances increased concentrations of E in the supernatant, Lat A pretreatment alone did not result in an E increase (Figure 1H). As a positive control, we employed the formylated tripeptide N‐formylmethionyl‐leucyl‐phenylalanine (fMLP), as this has long been known to strongly induce the secretion of vesicular components from neutrophils [38]. No alterations in neutrophil viability were observed under latrunculin A incubation (Figure S5). We also observed a robust release of E after neutrophil stimulation with latrunculin A and fMLP (Figure S6).

To corroborate our finding of CA release by neutrophils, we triggered exocytosis of FFN‐positive vesicles from neutrophils that had taken up FFN511 (Figure 1I). Indeed, 5‐HT (100 µm) induced degranulation and release of FFN511 from neutrophils. FFN511 imaging also allowed tracking of single vesicles during 5‐HT‐induced exocytosis, evidenced by a stepwise decrease of fluorescence intensity (Figure S7A). Additionally, we also employed FFN102, a pH‐sensitive FFN molecule with high affinity for DAT and VMAT2 [40]. The fluorescence of FFN102 is reduced inside vesicles due to their acidic pH, yet increases upon exocytosis into the extracellular space. Fluorescence in the FFN102 channel around the neutrophils increased after stimulation with 5‐HT (Figure S7B,C) compared to buffer control, which further supports that exocytosis of vesicle content occurs.

### 2.2 Imaging Catecholamine Exocytosis From Neutrophils in Real Time

**FIGURE 2:**
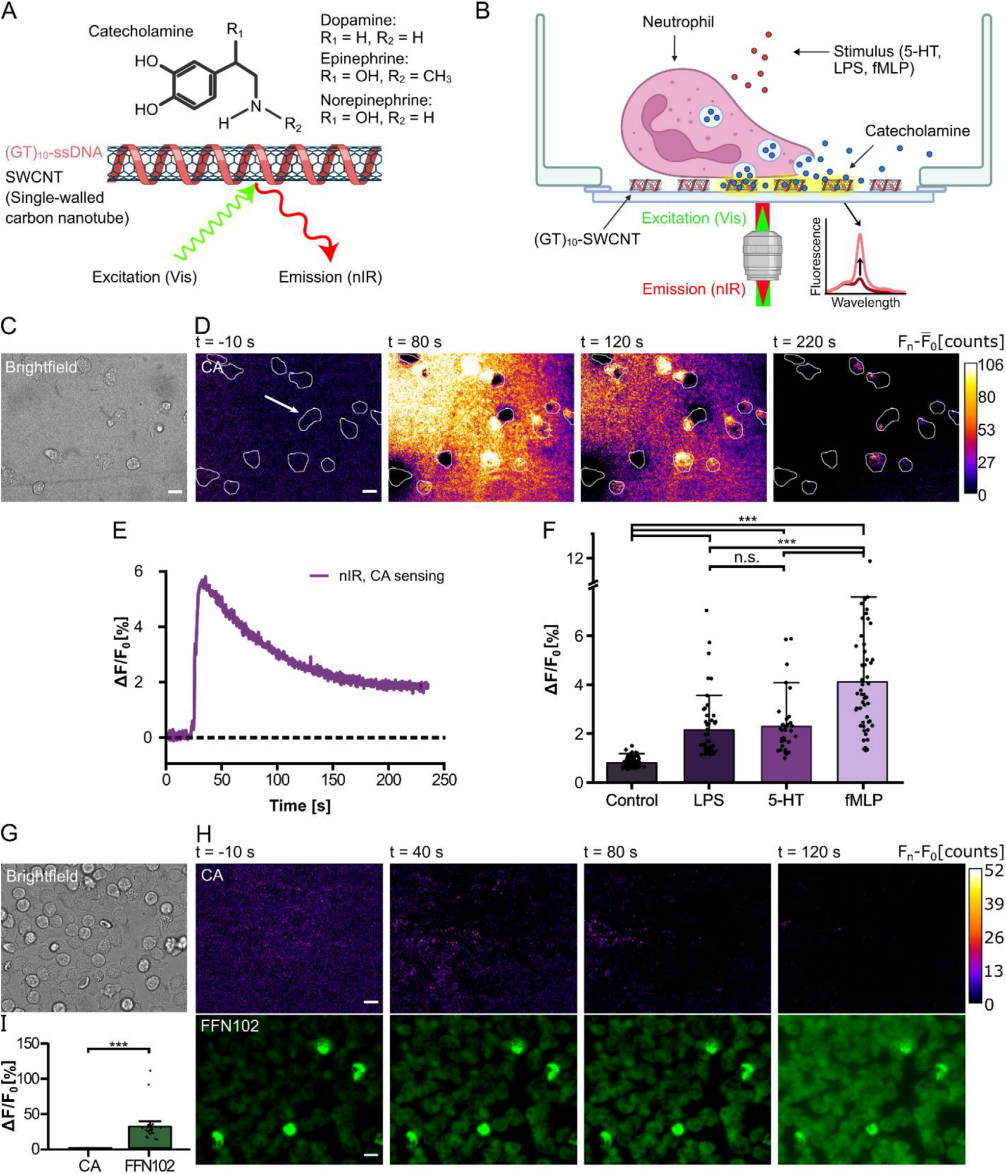
Exocytosis of catecholamines from neutrophils. (A) Schematic of the single‐walled carbon nanotube (SWCNT) based nanosensors used to image catecholamine (CA) release. Their nIR fluorescence (980 nm) increases by CA. (B) Schematic of the experimental setup, including immobilized nanosensors below adhering neutrophils. (C) Brightfield image and (D) nIR image sequence of neutrophils adhered on a nanosensor surface. The starting intensity is the mean pixel intensity value ($\bar{F}$_0_) of the 10 s before the addition of 5‐HT. 5‐HT (100 µm, *t* = 0) induces an increase in the fluorescence of nanosensors in localized regions corresponding to cell regions as well as everywhere by diffusion, indicating exocytosis of CAs. Scale bar is 10 µm. (E) Fluorescence response of nanosensors to CA exocytosis from a single neutrophil after stimulation with 5‐HT (around *t* = 20 s). The data was corrected for drift. (F) The fluorescence response of nanosensors (area under the cells) to CA exocytosis after stimulation with 100 µm 5‐HT, 100 nm N‐formylmethionine‐leucyl‐phenylalanine (fMLP), 25 µg/mL lipopolysaccharide (LPS), and a control with no treatment. (*n* = 4 donors, N ≥ 35 cells, mean ± SEM, Kruskal‐Wallis test, Mann‐Whitney tests: *p*‐value *** < 0.001, each dot is a single neutrophil). (G) Brightfield images (cells) and (H) nIR image sequence (CA nanosensors, top) and green channel (FFN102, bottom) of neutrophils incubated with FFN102 before and after addition of 5‐HT (100 µm). The background ($\bar{F}$_0_) is measured 10 s before the addition of 5‐HT. There is no fluorescence change in the nIR, indicating no CA release but the green channel (fluorescence increase in the extracellular space) indicates FFN102 release. Scale bar is 10 µm. (I) Maximum fluorescence response of the nanosensors (CA release) and green channel (FFN102 release) of neutrophils stimulated with 5‐HT (100 µm) after incubation with FFN102 (*n* = 2 donors, *N* = 30 cells mean ± SEM, unpaired *t*‐test: *p*‐value * < 0.05, each dot is a single neutrophil).

Transient release of small molecules from individual cells is difficult to capture with standard methods. To gain further real‐time knowledge about the release of CA from neutrophils, we employed a fluorescent nanosensor‐based approach to directly image CA release from neutrophils with high spatiotemporal resolution, as previously used for neurons [25, 28, 29]. For this purpose, we functionalized (6,5)‐chirality enriched nIR fluorescent SWCNTs with (GT)_10_ oligonucleotides (Figure 2A and Figure S8A). This DNA sequence renders SWCNTs sensitive to CA. These nanosensors increased their fluorescence (in solution) by > 50% in response to DA (100 µM) and other CA as expected (Figure S8B) [25, 28, 29]. Then they were homogenously physiosorbed on standard (glass) cell culture dishes (Figure S8C). Neutrophils were seeded on top of the nanosensors (Figure 2B). Pure DA (100 µm) increased the fluorescence of this thin sensor layer by ∼ 30 %, whereas buffer, ionomycin (Ca^2+^ ionophore), and 5‐HT (Figure S8D) did not affect the fluorescence intensity of the immobilized nanosensors. H_2_O_2_ also did not change the fluorescence (Figure S8E), which is important because reactive oxygen species such as H_2_O_2_ can be formed by neutrophils upon activation. These control experiments show that the nanosensor layer can specifically report local changes in CA concentration.

Corroborating our indirect findings with FFNs, 5‐HT stimulation of neutrophils increased the nanosensor fluorescence, which indicates CA exocytosis. Hot spots of CA release were associated with cell bodies, but the fast diffusion of CA quickly masked the cell bodies (Figure 2C,D). Release was followed by diffusion into the free extracellular space, and therefore single‐cell (fluorescence intensity under the cell body) traces were characterized by a steep increase followed by a slow decline of fluorescence intensity (Figure 2E). We observed variation in CA release amplitudes amongst donors and experiments (Figure S9), which shows that there is a certain biological heterogeneity. Again, the other previously tested degranulation stimuli (fMLP, LPS) also induced CA exocytosis (Figure 2F), however due to the significantly higher sensitivity of the nanosensors, this effect was visible without bolstering degranulation by latrunculin A as had been necessary for the CA ELISA (Figure 1D).

Neutrophils release a plethora of different molecules upon activation, including enzymes, cytokines, ROS, and small‐molecular signaling molecules. To exclude activation of the nanosensors by non‐CA molecules, we depleted CA in neutrophils by incubating them with FFN102 molecules. At high concentrations, FFN102s have been shown to deplete CAs from VMAT2‐positive vesicles and inhibit reuptake, without affecting other vesicular content [40]. As discussed above, FFN102 fluorescence increases once released into the extracellular space. FFN102‐treated neutrophils were then stimulated with 5‐HT and imaged simultaneously in the visible (Vis) (FFN102) and in the nIR (nanosensors) to detect endogenous CA (Figure 2G,H). While nIR fluorescence did not increase after 5‐HT stimulation in this scenario, FFN102 fluorescence significantly increased, indicating exocytosis of FFN102 after previous depletion of CA (Figure 2I) and underlining the specific fast and transient CA detection by the nanosensors.

### 2.3 Serotonin Triggers Intracellular Ca^2+^ Signals Followed by Catecholamine Exocytosis

**FIGURE 3:**
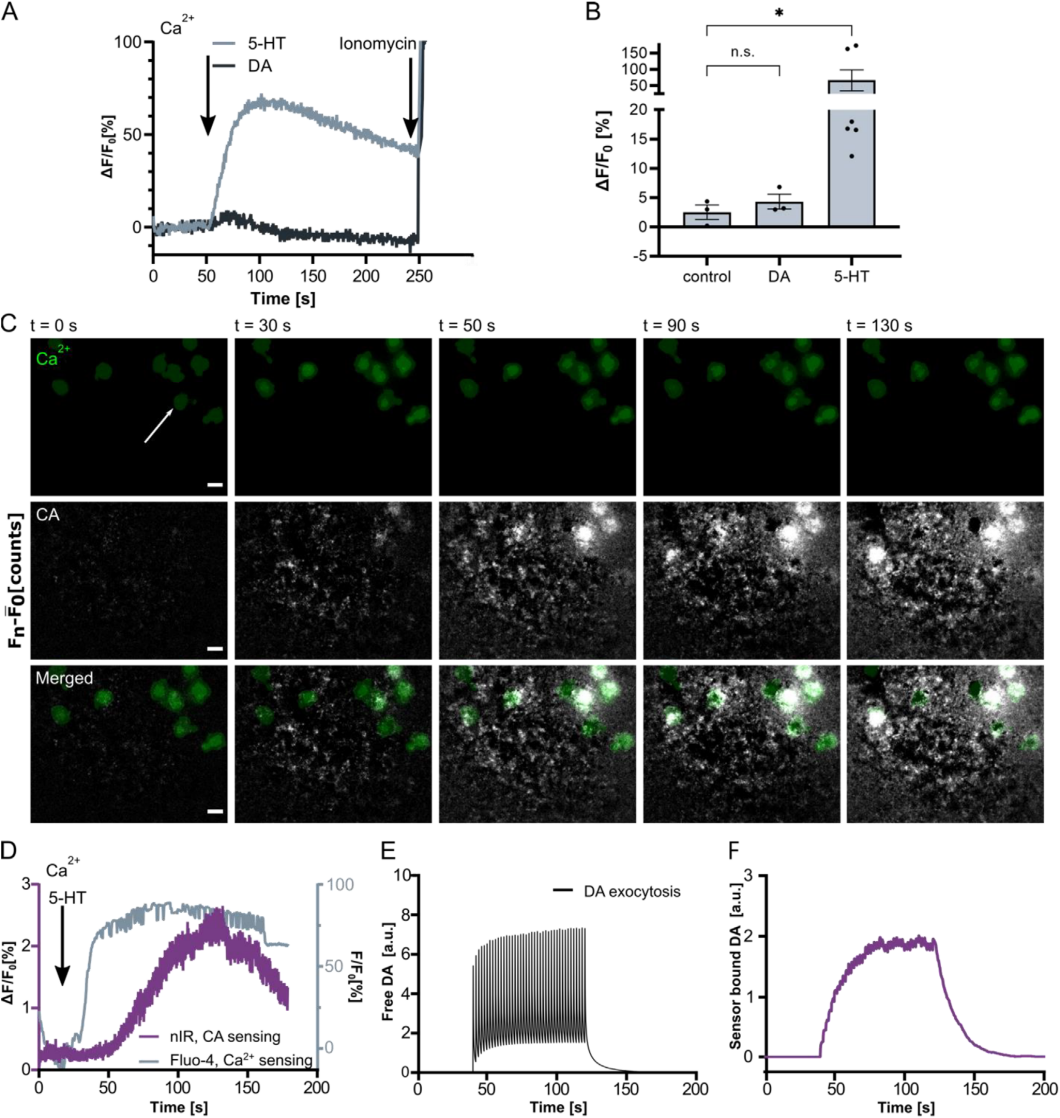
Serotonin triggers intracellular Ca^2+^ signals and catecholamine exocytosis. (A) Fluorescence signal of neutrophils incubated with Fluo‐4 AM over time. 5‐HT or dopamine (DA) (100 µm) is added at *t* = 50 s and ionomycin (5 µm) at *t* = 250 s. (B) Maximum fluorescence increase (calcium concentration change) for DA and 5‐HT (*n* ≥ 3 donors, mean ± SEM, Kruskal‐Wallis test with Dunn's multiple comparisons test: p value * < 0.05, each dot is a single experiment). (C) Neutrophils are adhered on nanosensor‐coated glass slides. Fluo‐4 AM (Ca^2+^) and nIR nanosensor channel (CA) before and after stimulation with 5‐HT (100 µM) at *t* = 0. All scale bars are 10 µm. (D) CA (nIR nanosensors, red) and Ca^2+^ (Fluo‐4, grey) time trace of an exemplary neutrophil (arrow in (D)). Fluorescence changes are the normalized intensity change under the cell outline (from the brightfield contour). (E) CA release and diffusion simulation: Simulated DA concentration in the area of a cell (diameter = 10 µm), after DA is released from several vesicles at the cell membrane in release events every 2 s over the course of 80 s (from *t* = 40 s to *t* = 120 s). (F) Corresponding simulated nanosensor fluorescence response, which is a convolution of concentration (E) and on‐off sensor kinetics.

Calcium signaling plays an important role in key neutrophil functions [41]. For example, exocytosis of gelatinase, specific, and azurophilic granules in neutrophils is triggered by a transient elevation in the intracellular calcium concentration. To better understand the processes accompanied by the 5‐HT‐induced CA exocytosis, we used the Ca^2+^ indicator Fluo‐4 AM [42]. Ionomycin‐induced Ca^2+^ influx was employed as a positive control (Figure S10). Indeed, 5‐HT but not DA induced a significant spike in the intracellular concentration of Ca^2+^ (>50 % increase) (Figure 3A,B). We employed the above‐mentioned nanosensor‐based sensing strategy to visualize exocytosis of CAs after stimulation with 5‐HT together with Ca^2+^ imaging (Figure 3C,D). Again, neutrophils were seeded on a nanosensor‐functionalized surface, and the Fluo‐4 AM fluorescence was imaged in the visible channel (GFP), while CA exocytosis was recorded simultaneously in the nIR channel (5 frames/s). 5‐HT increased the intracellular Ca^2+^ concentration, followed by a delayed (around 10–20 s) CA release (Figure 3C,D). This timescale is different from neuronal DA release, which typically happens within milliseconds (ms), suggesting that there are no or very few primed (docked and ready for release) vesicles in neutrophils as in neurons [29].

To better understand this process, we employed an in silico numerical diffusion simulation of the DA concentration, as an exemplary CA, in the immediate vicinity of a DA‐releasing neutrophil (Figure 3E). The simulated neutrophil released DA over the course of 80 s from several vesicles (Figure 3E and Tables S4, S5). Importantly, the measurable fluorescence signals are not the concentration but the convolution/overlay between concentration and sensor kinetics. Therefore, we also simulated the corresponding amount of sensor‐bound DA (Figure 3F), accounting for on‐off kinetics (Tables S4, S5). The qualitative similarity to Figure 3D indicates that CAs are released from several vesicles over several 10 s around the cell as opposed to other simulated scenarios, such as single release events (Figure S11 and Table S5).

### 2.4 Serotonin‐Induced CA Release Requires Serotonin Receptors and Can be Elicited by Platelet‐Neutrophil Interactions

**FIGURE 4:**
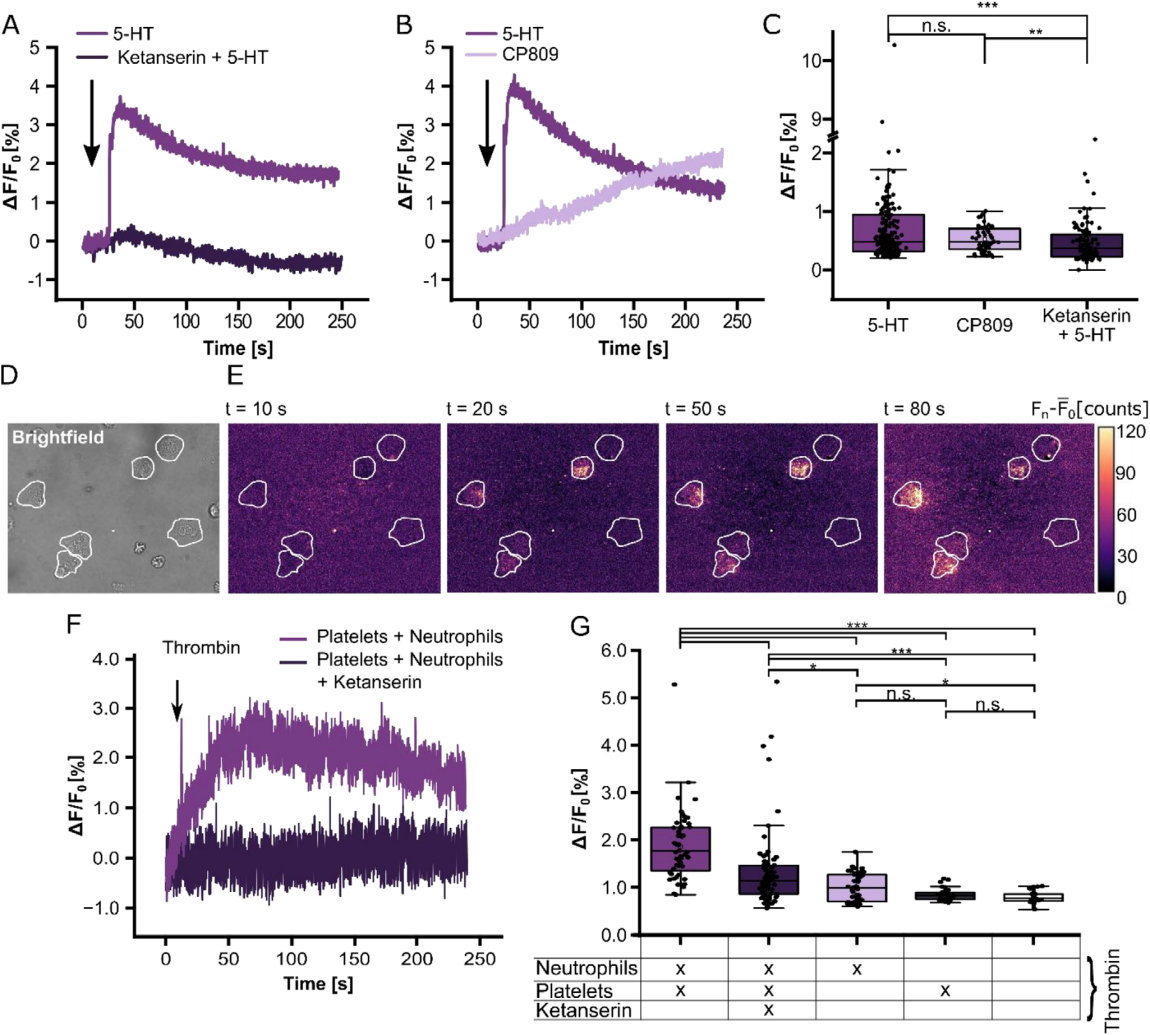
Catecholamine exocytosis is serotonin receptor dependent and triggered by activated platelets. (A) Nanosensor signal from an exemplary neutrophil stimulated with 5‐HT (100 µm) and treated with and without 5‐HT antagonist ketanserin (100 µm). (B) Nanosensor signal from an exemplary neutrophil stimulated with 5‐HT or 5‐HT agonist CP809. (C) Maximum nanosensor fluorescence increase of neutrophils after stimulation with 5‐HT (100 µm), treated with ketanserin (100 µm) or CP809 (1 µm). (*n* ≥ 5, mean ± SEM, Kruskal‐Wallis test and Mann‐Whitney tests: *p*‐value *** < 0.001, ** < 0.01, each dot is a single neutrophil). (D) Brightfield image and (E) nIR image sequence (right) of neutrophils and platelets adhered on a nanosensor‐coated glass surface. nIR images are recorded before and after stimulation of platelets with thrombin (1 Unit/mL). The outlines indicate the shapes of neutrophils. (F) Exemplary fluorescence responses of nanosensors to neutrophil + adhered platelets + thrombin and neutrophil + adhered platelets + 100 µm ketanserin + thrombin. (G) Maximum fluorescence‐increase of nanosensors for neutrophil + adhered platelets + thrombin, neutrophil + adhered platelets + 100 µm ketanserin + thrombin, neutrophil + thrombin, adhered platelets + thrombin, and a control without cells + thrombin (*n* = 4 donors, *N* ≥ 45 cells, mean ± SEM, Kruskal‐Wallis test and Mann‐Whitney tests: *p*‐value * < 0.05, *** <0.001, each data point corresponds to a single neutrophil).

To investigate whether 5‐HT‐induced exocytosis is specifically 5‐HT receptor mediated, we pre‐treated neutrophils with the 5‐HT2 selective antagonist ketanserin [43]. After neutrophils adhered to the nanosensor‐coated surface, ketanserin (100 µm) was added for 2–5 min before exposing them to 5‐HT or ionomycin, as a control. Ketanserin‐treated neutrophils did not release CA when exposed to 5‐HT (Figure 4A and Figure S12A,B) but, as expected, ionomycin still increased fluorescence at localized regions around cells corresponding to CA release (Figure S12A—C). The 5‐HT receptor agonist 2‐[(3‐chlorophenyl)methoxy]‐6‐(1‐piperazinyl)‐pyrazine (CP809) also induced exocytosis of CA but with slower kinetics (Figure 4B,C and Figure S12D,E). These results indicate that 5‐HT2 engagement is necessary for serotonin‐induced CA exocytosis in neutrophils.

While about 90% of the body's 5‐HT is produced in the gut in enterochromaffin cells, a significant portion then enters the bloodstream, where it is rapidly taken up by platelets, which then constitute the major source of 5‐HT in the periphery and can release this content in a context‐dependent manner [44, 45]. Because of the previously well‐documented importance of 5‐HT in platelet‐neutrophil interactions, we next studied platelet‐neutrophil interactions by seeding platelets and neutrophils together on nanosensor‐coated surfaces [45]. Neutrophils exposed to thrombin‐activated platelets released CA (Figure 4D–G). In contrast, thrombin alone as well as thrombin + platelets without neutrophils did not show any change in fluorescence (Figure 4F). To exclude that CA‐exocytosis from neutrophils was induced by an impact of thrombin on neutrophils, neutrophils were inhibited with ketanserin before incubation with thrombin‐activated platelets. This treatment abolished CA‐release, corroborating that the interaction of activated platelets and neutrophilic granulocytes activates exocytosis of CA by neutrophils via a 5‐HT‐dependent mechanism (Figure 4G).

### 2.5 Catecholamines Modulate Neutrophil Functions as Well as Platelet Aggregation

**FIGURE 5:**
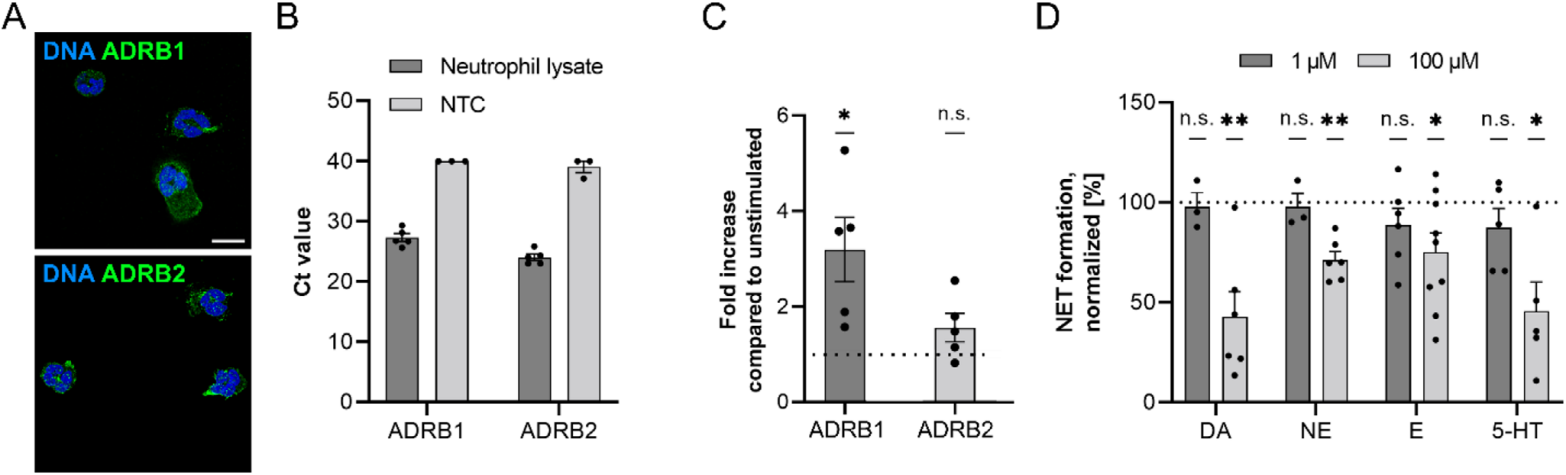
Paracrine catecholamine signaling impacts NETosis. (A) Neutrophils stained for β1‐adrenergic receptor (ADRB1, green) or β2‐adrenergic receptor (ADRB2, green) and DNA (blue). Scale bar is 10 µm. (B) Both receptors are found at the transcriptional level as determined by qPCR (*n* = 5 and *n* = 3 donors, mean ± SEM, NTC: no template control). (C) The expression of ADRB1 but not ADRB2 increases after stimulation of neutrophils with 50 ng/mL LPS for two hours (*n* = 5 donors, mean ± SEM, one‐sample *t*‐test). (D) Effect of CAs as well as 5‐HT on NETosis in neutrophils stimulated with 5 nm PMA. PMA stimulated controls = 100 %, (*n* ≥ 3, mean ± SEM, one‐sample *t*‐test, or one‐sample Wilcoxon test: *p*‐value * < 0.05, ** < 0.01).

**FIGURE 6:**
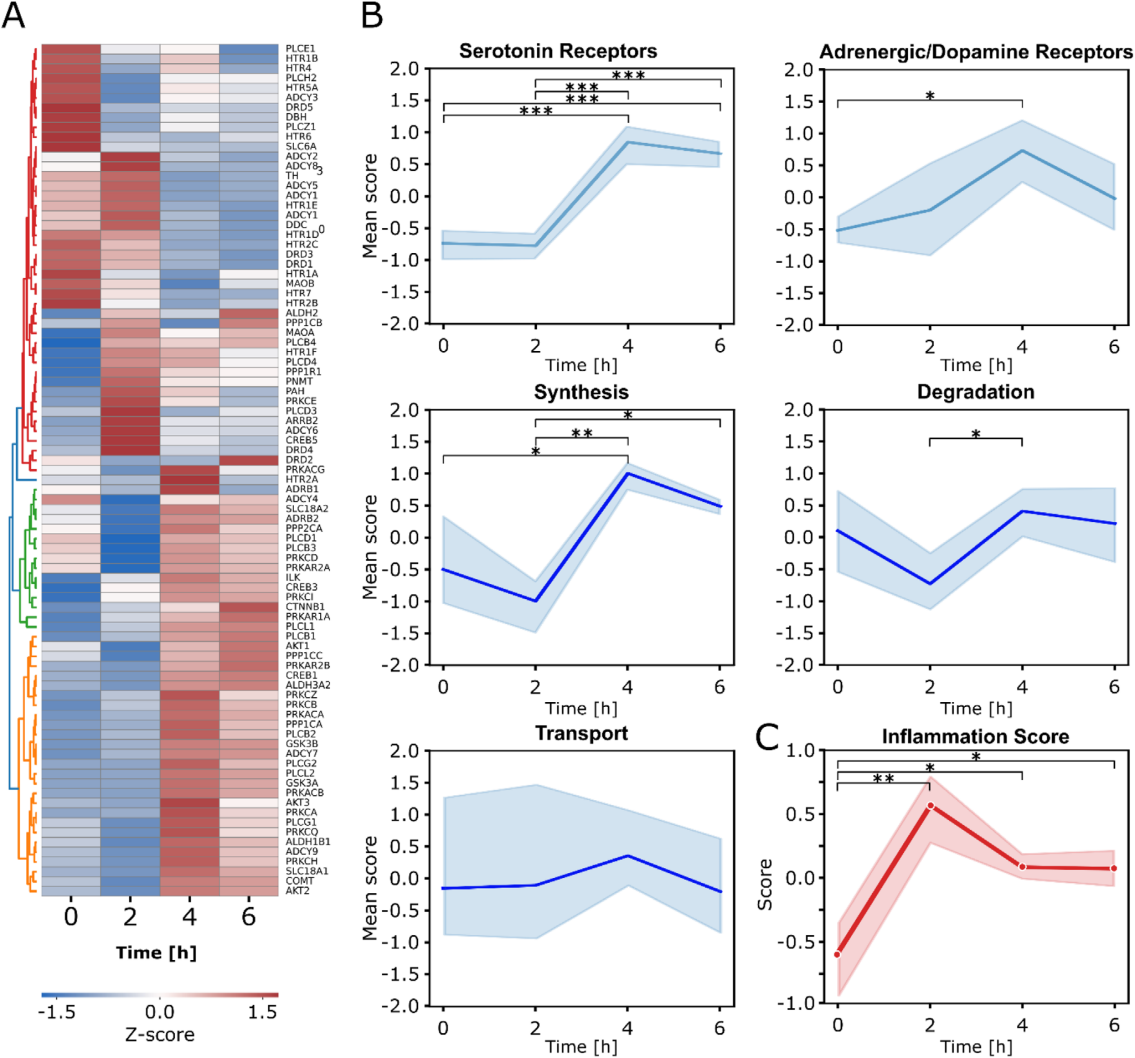
Neutrophils transcriptionally regulate monoaminergic signaling pathways during systemic inflammation. (A) Heatmap of z‐scored gene expression profiles for 87 curated monoaminergic genes from human neutrophils isolated at 0, 2, 4, and 6 h after LPS administration (GSE35590, *n* = 4 per condition). Genes were hierarchically clustered using Euclidean distance and average linkage. (B) Mean z‐scored expression of gene sets grouped by functional category: serotonin receptor expression, dopamine‐ and adrenoreceptor expression, CA synthesis, CA degradation, and transport (*n* = 4 donors, mean ± 95% confidence intervals, computed from bootstrapped means across genes within each category, Tukey's post hoc test: p‐value * < 0.05, ** < 0.01, ***, <0.001). (C) Inflammation score calculated from the average z‐scores of 26 inflammation‐related genes (e.g., IL1B, TNF, S100A8/A9, MPO, CXCR1/2 (Table S8)). (*n* = 4 donors, mean ± 95% confidence intervals, *p*‐value * < 0.05, ** < 0.01).

As explained above, tight control of neutrophil functions is essential to prevent damage to the host. For this reason, we next tested the hypothesis that neutrophils use CAs to down‐regulate (excessive) inflammatory activities. CAs have an affinity to bind a large variety of receptors, including different DA and adrenergic receptors. The expression of β‐adrenergic receptors on neutrophils, which are able to bind E, NE, and DA, has long been established [16, 46, 47]. We here verified the presence of both β‐adrenoreceptors, ADRB1 and ADRB2, on the protein level by immunofluorescence staining (Figure 5A) and by qPCR on the mRNA level (Figure 5B). Interestingly, ADRB1 expression increased in neutrophils incubated ex vivo with 50 ng/mL LPS for two hours (Figure 5C) and both β‐adrenergic receptors were upregulated in volunteers receiving a bolus of iv LPS (Figure 6A) at 2 and 4 h, respectively, pointing to a dynamic role of CA signaling with adaptive responsiveness to ß‐adrenoreceptor‐signaling in neutrophils under inflammatory conditions.

To determine the effect of CA on a prototypical, highly inflammatory neutrophil function, we next examined the effect of various monoamines (E, NE, DA, 5‐HT) on the rate of NETosis with and without stimulation with phorbol‐12‐myristate‐13‐acetate (5 nm PMA), a commonly used stimulus of NET formation. All monoamines reduced NET formation compared to the control group in a concentration‐dependent manner (Figure 5D and Figure S13). In the case of 5‐HT, this may be caused directly or indirectly by stimulating CA exocytosis.

Given the now established importance of activated platelets in neutrophil‐related CA release, we next aimed to contextualize the impact of CA on platelet behavior. Direct pro‐aggregatory effects of catecholamines on platelets are well documented [48, 49], and our experiments corroborated these findings: in optical aggregometry [50], all tested catecholamines accelerated thrombin‐induced aggregation (as evidenced by smaller K_m_ values; Figure S14A), and NE and E also increased maximal aggregation amplitudes (Figure S14B). These data illustrate that platelets are highly responsive to catecholamines and are consistent with the plausibility of a paracrine circuit [48, 49, 50].

### 2.6 Monoaminergic Signaling Pathways are Transcriptionally Regulated in Human Endotoxemia

As shown above, 5‐HT but also LPS induce CA release from neutrophils (Figures 1D and 2F). Of note, extensive connections between inflammation and coagulation have previously been reported, with platelets, neutrophils, and NETs as key players [51]. Our previous results point to an anti‐inflammatory and pro‐coagulant effect of CA, which may play a role in the course of bacterial infections and septicemia‐HT initial activation of the immune system may require a downregulation of neutrophil pro‐inflammatory functions to limit tissue damage, and are very often associated with pro‐coagulant states [51].

To investigate the transcriptional dynamics of monoaminergic signaling in neutrophils during systemic inflammation, we analyzed gene expression profiles from a human model (GSE35590, Table S6) [52]. This dataset stems from an intravenous LPS challenge in healthy human volunteers, designed to induce rapid systemic inflammation. Blood samples were collected at baseline and multiple post‐LPS time points (e.g., 2, 4, 6 h).

To account for baseline expression differences between genes, the expression matrix was z‐score normalized across time points (Figure 6A). Unsupervised clustering revealed several coherent co‐regulated gene modules with distinct temporal trajectories. Cluster 1 (*n* = 27 genes, orange) included second messenger signaling components such as ADCY7 (adenylyl cyclase type 7), GSK3B (glycogen synthase kinase‐3 beta), and PRKCA (protein kinase C alpha), as well as the monoamine transporter SLC18A1. These genes showed declining expression patterns. Cluster 2 (*n* = 15 genes, green) contained regulatory isoforms of protein kinase A and C, together with adrenergic receptors (ADRB1, ADRB2), and displayed moderate, variable expression trends. One of the prominent upregulated clusters (Cluster 3, *n* = 44 genes, red) featured serotonin receptors (HTR1B, HTR2A, HTR4, HTR6) alongside key biosynthetic enzymes such as TH and DOPA decarboxylase (DDC), supporting the idea of a coordinated transcriptional program that enhances serotonin signaling capacity.

To summarize expression changes at the pathway level, we calculated average z‐score trajectories for manually curated functional categories (Table S6). The time courses (Figure 6B) of five monoaminergic categories (serotonin receptors, ß‐adrenergic receptors, and DA receptors, synthesis, degradation, and transport) revealed a biphasic regulatory pattern. An early phase with a trend toward reduced expression of degradation and synthesis of CA, followed by a later significant upregulation of serotonin receptors and enzymes needed for CA synthesis and degradation. Furthermore, adrenoreceptors and dopamine receptors together displayed a robust and sustained activation with a significant upregulation at 2 h and a peak between 4 and 6 h (Figure 6B), suggesting an upregulation of CA sensing in order to dampen inflammatory responses. It should be noted that cluster 3 in Figure 6A had emerged through unsupervised clustering and consisted predominantly of receptor genes, supporting the function‐based classification in Figure 6B.

To further dissect receptor regulation, we stratified serotonin receptors by their annotated functional mode—activating vs. inhibiting (Table S7) —and examined their temporal dynamics (Figure S15A). Both activating and inhibiting receptors were transcriptionally induced, although subtle differences in timing and amplitude were noted. These patterns suggest that the balance of excitatory and inhibitory serotonergic signaling may be dynamically adjusted during inflammation via selective transcriptional control.

In contrast, CA transporters did not show any significant regulation of expression in line with a constitutive readiness to uptake CA without the need of transcriptional regulation (Figure S15B). This is in line with our previous results showing rapid FFN uptake upon exposure of neutrophils to FFNs (Figure 1G) and their capability to pass on CA like molecules to neighboring neutrophils (Figure S16).

To place these findings in a broader inflammatory context, we computed an aggregate inflammation score based on a curated set of 26 genes involved in cytokine signaling, oxidative burst, and neutrophil activation (e.g., IL1B, IL6, TNF, S100A8/A9, MPO, ELANE, CXCR1/2)(Table S8). Mean z‐scores across these genes were calculated per donor and time point. As shown in Figure 6C, the inflammation score sharply increased at 2 h post‐LPS, with differences relative to baseline (*p* < 0.01), followed by a gradual decline toward 6 h, albeit still elevated (*p* < 0.05). These dynamics confirm that the monoaminergic signature emerges in parallel with systemic neutrophil activation.

Together, these results suggest a temporally structured and biologically coordinated transcriptional program in neutrophils that may contribute to priming the catecholaminergic system during the inflammatory response.

## 3 Discussion

Neutrophils are among the fastest and most numerous responders to acute infection and play a key role in the coagulation cascade, bridging inflammation and coagulation. The potentially highly destructive activity of neutrophils requires a tight regulation of these effector functions, both locally and temporally. Catecholamines (CAs) such as epinephrine (E), norepinephrine (NE) and dopamine (DA) are small molecular compounds that serve as neurotransmitters in the brain and signaling molecules in the periphery. While anti‐inflammatory effects of CA on neutrophils have previously been established, if and how neutrophils synthesize endogenous CAs, store and release CAs and regulate their CA machinery upon stimulation has so far remained elusive.

We show that the dopaminergic machinery known from neurons is present in neutrophils. We not only confirm [53] that TH, the rate‐limiting enzyme for catecholamine synthesis, is present on both the protein and mRNA level (Figure 1A), but also provide evidence that neutrophils synthesize catecholamines ex vivo after activation with either LPS or 5‐HT (Figure 1). In these experiments, intracellular catecholamine levels increased despite the absence of exogenous catecholamines, making reuptake an unlikely explanation and supporting catecholamine production by neutrophils.

In addition, all three main catecholamines (DA and to a lesser extent E and NE) as well as metabolic (degradation) products could be detected inside neutrophils (Figure 1D), indicating the presence of an active catecholamine metabolic system. Notably, this increase in intracellular catecholamines is unlikely to be explained by reduced degradation alone, as transcriptomic data from the human LPS challenge indicate that genes involved in both catecholamine synthesis and degradation are dynamically regulated during inflammation, arguing against a model of passive catecholamine accumulation due to impaired breakdown and instead supporting an actively regulated catecholaminergic system in neutrophils.

CA trafficking, including uptake and exocytosis was studied by using FFNs (Figure 1E), a tool established in neuronal biology but, to our knowledge, not previously applied to neutrophils or other immune cells [31, 40]. Our results reveal that VMAT2 specifically contributes to catecholamine uptake (Figure 1F,G), consistent with the presence of intracellular vesicle‐like compartments in neutrophils (Figure 1A).

The very rapid FFN uptake by neutrophils within seconds (Figure 1F,G) suggests that these cells have a high intrinsic capacity for catecholamine uptake. Transcriptomic analysis from the controlled human LPS challenge model (GSE35590) revealed that genes implicated in CA transport remain robustly and constitutively expressed throughout the time course (Figure 6B), indicating that this uptake propensity is pre‐existing and does not rely on de novo transcription. In contrast, genes involved in CA synthesis and degradation showed dynamic regulation (Figure 6A,B), pointing to a transcriptionally adjustable component of the neutrophil catecholaminergic system.

Interestingly, we identified fMLP, LPS, and serotonin (5‐HT), all of which are known inducers of neutrophil degranulation [35], as triggers of CA release from neutrophils (Figures 1H and 2F). In parallel, 5‐HT and LPS increased intracellular CA content ex vivo (Figure 1D), consistent with induced CA synthesis under inflammatory conditions. 5‐HT is of particular interest in neutrophil biology because it is stored and released by platelets, and platelets and neutrophils engage in close contact during coagulation and thrombosis. Together, these observations point to a 5‐HT–driven axis that functionally links inflammatory stimuli, platelet‐derived signals, and neutrophil catecholamine release.

Because FFNs report vesicular content only indirectly, we next used nIR fluorescent nanosensors specifically tailored for CA detection [54], which offer substantially higher spatiotemporal resolution. With this approach, we directly visualized fast and transient CA release events from neutrophils (Figure 2A–F). In neurons, vesicular CA concentrations are estimated to be in the range of approximately 300 mm [55]. Exocytosis of such high‐concentration cargo produces steep, short‐lived extracellular catecholamine peaks that dissipate rapidly by diffusion. These dynamics are difficult to resolve with conventional biochemical approaches and likely explain why CA release from neutrophils has remained unexplored. Our nanosensor findings were independently supported by experiments using the pH‐sensitive FFN102, whose fluorescence increases upon release into the extracellular space after 5‐HT stimulation (Figure 2G–I).

Several studies indicate that Ca^2^^+^ mobilization is an important regulator of neutrophil degranulation [56, 57]. To further dissect the mechanism of CA release, we simultaneously visualized intracellular Ca^2^^+^ dynamics and extracellular CA exocytosis using a custom‐built dual nIR/Vis fluorescence microscope setup developed in our laboratory. We found that 5‐HT induces an intracellular Ca^2+^ rise followed by delayed CA release (Figure 3A–D) and that this process requires 5‐HT2 receptor engagement (Figure 4A,C).

The markedly slower kinetics of CA release in neutrophils (>10 s) compared to the millisecond‐scale fusion events in neurons suggest that the responsible vesicular compartments operate under immune cell–specific fusion programs rather than neuronal paradigms (Figure 3E,F). This interpretation is further supported by our in silico diffusion modeling, which indicates that the temporal profile of sensor‐detected CA is best explained by sequential release from multiple vesicles over several tens of seconds rather than by rapid single‐vesicle fusion events (Figure 3E,F). Neutrophils harbor several granule and vesicle classes with differing fusion thresholds, and CA‐containing compartments may therefore require integration of multiple inflammatory cues before exocytosis. Consistent with this, distinct stimuli (fMLP, LPS, 5‐HT) triggered CA release to varying degrees (Figure 1H and 2F), supporting a modulatory role of CAs within the broader neutrophil activation program. These features also raise the possibility that neutrophil subpopulations differ in their capacity for CA release depending on maturation state, adhesion, or microenvironmental context. Indeed, neutrophil subgroup responses might be reminiscent of the functional plasticity observed in neuronal systems. Future studies will be needed to determine how such heterogeneity shapes catecholaminergic responses in vivo.

Of note, our data further place functional constraints on the vesicular system underlying catecholamine handling in neutrophils. Catecholamine release occurred within seconds to minutes following stimulation and was closely associated with intracellular calcium transients, indicating that the responsible compartments must be rapidly mobilizable and responsive to calcium‐dependent signaling. In addition, catecholamine stability requires a mildly acidic and non‐oxidative environment, suggesting that these vesicles provide appropriate physicochemical conditions for storage. Notably, release could be triggered by a single inflammatory stimuli without prior priming, pointing to a system that is readily accessible within the neutrophil activation program. Together, these observations are consistent with a dynamic and readily mobilizable vesicular system. However, the precise identity and trafficking pathways of these compartments remain to be defined and will require further dedicated investigation.

Platelet–neutrophil interactions are key elements of immunothrombosis and shape local inflammatory responses. To investigate the putative interplay between neutrophils and platelets in a more physiological scenario, we brought neutrophils in contact with thrombin‐activated platelets, as thrombin is known to induce 5‐HT release from platelets [58]. Indeed, in this more physiological setting, thrombin‐activated platelets robustly triggered CA release from adjacent neutrophils, and this effect was abolished by 5‐HT2 receptor blockade (Figure 4D–G). These experiments provide direct evidence that platelet‐derived 5‐HT can elicit catecholamine exocytosis from neutrophils, establishing a monoamine‐sensitive interaction between these cell types.

We next examined whether catecholamines feed back onto neutrophil effector functions. All tested monoamines (E, NE, DA, 5‐HT) reduced PMA‐induced NET formation in a concentration‐dependent manner (Figure 5D), consistent with an anti‐inflammatory role of catecholamines in neutrophils. This aligns with previous reports showing β‐adrenergic, catecholamine‐mediated dampening of other neutrophil functions, including reduced phagocytosis, downregulation of CD11b and CD18, and decreased ROS release [59, 60]. These findings indicate that catecholamines not only are released by neutrophils but can also modulate neutrophil activation states, suggesting the existence of a local, monoamine‐sensitive regulatory circuit.

**FIGURE 7:**
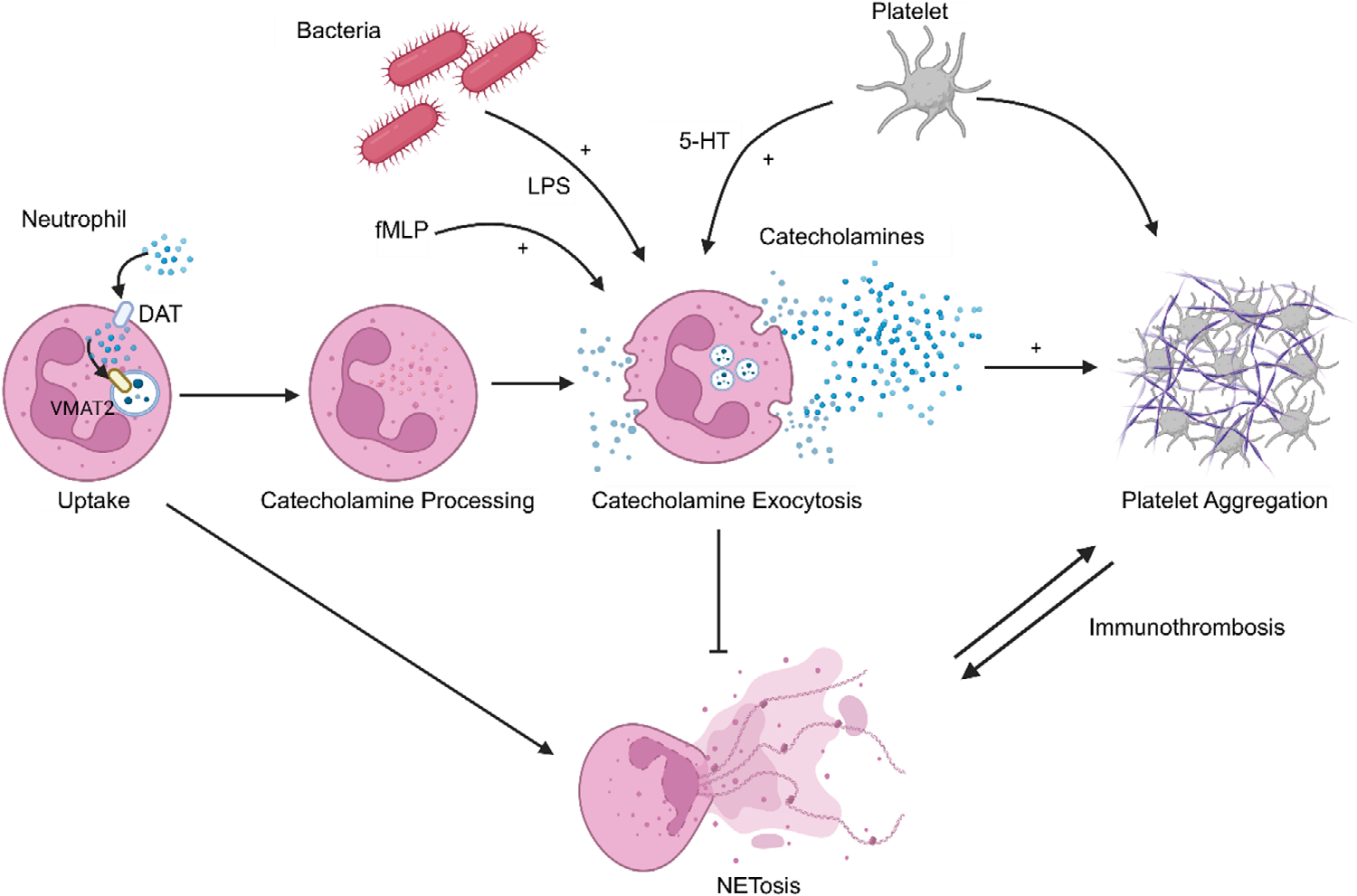
Model for catecholamine‐mediated immunomodulatory feedback loop between neutrophils and platelets. Neutrophils synthesize or take up catecholamines (CAs) via DAT and package them into vesicles using VMAT2. Certain substances, including serotonin released by activated platelets trigger exocytosis of CAs, which enhances platelet aggregation and inhibits NETosis.

Moreover, catecholamines also influence platelet activation. In our aggregometry experiments, exogenously added catecholamines accelerated thrombin‐induced platelet aggregation and increased overall aggregation responses (Figure S14), in line with previous reports on catecholamine‐sensitive platelet activation. Together with our finding that activated platelets trigger serotonin‐dependent catecholamine release from neutrophils (Figure 4), these observations support the concept that monoamine signaling can contribute to reciprocal platelet–neutrophil communication during thromboinflammatory processes. In this framework, locally released catecholamines may act in an autocrine or paracrine manner within inflammatory microenvironments (Figure 7).

We corroborated earlier reports that neutrophils express the β‐adrenergic receptors ADRB1 and ADRB2 [15, 61] and found that ADRB1 expression increases ex vivo two hours after LPS stimulation (Figure 5A–C), indicating dynamic regulation of catecholamine responsiveness during inflammation. Analysis of neutrophil transcriptomes from the human LPS challenge model (GSE35590) further refined this picture: monoaminergic pathways displayed temporally structured regulation, with stable transporter expression but delayed induction of serotonin receptors and catecholamine biosynthetic enzymes (Figure 6A,B). These data support a biphasic regulatory model in which an early phase of attenuated synthesis and degradation is followed by a later transcriptional program that enhances receptor signaling and biosynthetic capacity. Notably, these monoaminergic signatures rose after the peak of the general neutrophil inflammation score (Figure 6C), suggesting a counter‐regulatory adaptation that enables neutrophils to modulate their functional state during sustained immune activation.

It is important to consider these findings within the broader physiological context. Systemic exposure to pathogens, LPS, or other severe stressors—such as strenuous physical exertion—elicits a rapid neuroendocrine response that includes catecholamine release from chromaffin cells. Such global, sustained elevations of circulating catecholamines differ fundamentally from the transient, spatially confined catecholamine peaks released locally by neutrophils. Systemic catecholamine surges have long been associated with an overall dampening of inflammatory responses, as observed in clinical settings where catecholamines are administered for cardiovascular support [62]. In contrast, we propose that neutrophil‐derived catecholamines may operate predominantly in localized inflammatory niches, such as infected tissues or sites of immunothrombosis, where they could initially permit robust activation yet subsequently promote a shift toward a more restrained, anti‐inflammatory state. The delayed transcriptional induction of monoaminergic genes during human endotoxemia (Figure 6) supports a model in which systemic inflammation sensitizes neutrophils to local autocrine or paracrine catecholamine cues.

Interestingly, catecholaminergic signaling in neutrophils may also be influenced by psychological stress. A recent study reported increased TH expression in neutrophils following psychological trauma and identified a β‐adrenergic–dependent impairment of bone fracture healing under these conditions [63]. Although that study did not demonstrate catecholamine production or release by neutrophils, it aligns with our findings by implicating neutrophils in neuroimmune crosstalk pathways. Together, these data highlight a previously underappreciated role of the neutrophil catecholaminergic system at the interface of inflammation, immunity, coagulation, and tissue repair—an axis that warrants further investigation.

## 4 Conclusion

Catecholamines (CAs) are well established as neurotransmitters and neuroendocrine mediators. Here, we show that human neutrophils possess the molecular machinery for CA synthesis, uptake, vesicular storage, and release, and that CAs modulate central neutrophil and platelet functions. By integrating CA‐sensitive nIR single‐walled carbon nanotube nanosensors, fluorescent false neurotransmitters (FFNs), and simultaneous Ca^2^^+^ imaging, we resolved rapid CA uptake and release dynamics with high spatiotemporal precision. Notably, FFNs—previously used almost exclusively in neuronal systems—enabled visualization of vesicular CA handling in neutrophils, while dual nIR/visible imaging linked intracellular Ca^2^^+^ signals to subsequent CA exocytosis. Transcriptomic profiling during systemic inflammation further demonstrated that monoaminergic pathways in neutrophils are dynamically regulated, supporting a broader role for CAs in immune modulation. Our data indicate that catecholamine signaling establishes a locally acting, concentration‐dependent feedback mechanism that shapes neutrophil behavior in inflammatory microenvironments. In this respect, neutrophil CA signaling shows conceptual parallels to volume transmission in the central nervous system. Overall, our findings support a model in which the immune system uses localized CA release as a rapid and spatially confined immunoregulatory mechanism.

## 5 Materials and Methods

### 5.1 Isolation of Human Neutrophils From Whole Blood

This study was approved by the Ethics Committee Westfalen‐Lippe (approval number 2021‐657‐f‐S). Before donating blood, fully informed consent of each donor was obtained.

Neutrophils were isolated from the whole blood of healthy donors by the density gradient separation method. Briefly, fresh blood was collected into EDTA blood collection tubes (Sarstedt, Germany). Whole blood was layered on Histopaque 1119 (Sigma‐Aldrich, United States) and centrifuged at 1100 x g, for 21 min at room temperature with breaks off. The neutrophil layers (the third layer mainly containing density gradient and the fourth layer mainly containing neutrophils) were collected and diluted in Hank's Balanced Salt Solution (HBSS, Sigma‐Aldrich, United States) without Ca^2+^ and Mg^2+^. The cell suspension was centrifuged at 400 x g, for 10 min at room temperature. The supernatant was discarded, and the cell pellet was resuspended in HBSS, layered on top of a five‐step Percoll gradient (GE Healthcare, United States), and centrifuged at 1100 x g for 21 min at room temperature with breaks off. The neutrophils were collected from the fourth layer and a bit from the layers above and below the fourth. The neutrophils were diluted in HBSS and centrifuged at 400 x g, for 10 min at room temperature. The pelleted neutrophils were resuspended in RPMI 1640 cell medium (Gibco, United States) containing 10 mm HEPES (Sigma‐Aldrich, United States), and 0.5 % fetal calf serum (FCS, heat‐inactivated at 56°C for 30 min, Gibco, United States) as needed.

For PCR and ELISA assays as well as FFN uptake experiments, neutrophils were isolated from whole blood using the EasySep Direct Human Neutrophil Isolation Kit (Stemcell Technologies, Canada) according to the manufacturer's instructions. The isolated neutrophil suspension was pelleted by centrifugation at 400 x g, for 10 min at room temperature, and the pellet was taken up in RPMI 1640 cell medium containing 10 mM HEPES, and 0.5 % fetal calf serum (FCS, heat‐inactivated at 56°C for 30 min as needed. We confirmed the purity of neutrophils by a cytospin assay and staining using the Panoptical Fast Staining Kit (Carl Roth, Germany). We maintained neutrophil purity > 95 % of total isolated cells (without erythrocytes). Neutrophils were used for experiments immediately after isolation.

### 5.2 Immunofluorescence Staining

A total of 1–2 × 10^5^ cells in 500 µL RPMI 1640 with 10 mm HEPES, 0.5 % FCS were seeded on round glass coverslips and left to adhere for 30–180 min at 37°C, 5 % CO_2_.

The adhered neutrophils were fixed by adding paraformaldehyde (PFA, biotium, United States or Morphisto, Germany) at a final concentration of 2 % for 15 min at room temperature. Cells were washed twice with PBS (Sigma‐Aldrich, United States) and then permeabilized using PBS with 0.1 % Triton X 100 (Sigma‐Aldrich, United States) for 10 min at room temperature. After two further washing steps with PBS, neutrophils were blocked with PBS containing 0.5%–10 % bovine serum albumin (BSA, Capricorn Scientific, Germany) for 40 min at room temperature. Fixed cells were incubated with primary antibodies (Table S1) or the respective isotype control in PBS containing 0.5%–1% BSA overnight at 4°C. Cells were washed three times with PBS and subsequently incubated with secondary antibody (Table S1) in PBS containing 0.5%–1 % BSA for 1 h at 37°C. After three further washing steps, the samples were stained with Hoechst 33342 (Invitrogen, United States) for 15 min and mounted using fluorescence mounting medium (Agilent, United States). The stained neutrophils were imaged with an Eclipse Ti2 microscope (Nikon Instruments, Japan) equipped with an Orca Fusion BT camera (Hamamatsu Photonics, Japan), an Axiovert 200 microscope (Zeiss, Germany) with a CoolSNAP ES camera (Photometrics, United States) or an LSM800 Airyscan confocal microscope (Zeiss, Germany).

For staining of vesicular monoamine transporter 2 (VMAT2) the adhered cells were carefully washed twice with warm PBS and fixed with ice‐cold methanol (VWR Chemicals, United States) for 10 min at 4°C. The cells were washed twice with PBS and then blocked for 3 h with 1 % BSA (Carl Roth, Germany) in PBS at room temperature. The antibodies against VMAT2 as well as the isotype (Table S1) were incubated with the cells overnight at 4°C in 1% BSA solution. The following day, the cells were washed twice with PBS. Subsequently, the second antibodies Alexa Fluor 555 (1:1000, Invitrogen, United States) (Table S1) was incubated with the cells for 1 h at room temperature in 1 % BSA solution. The cells were then washed twice for 10 min with PBS, twice with 0.1 % polyoxyethylene sorbitan monolaurate (Tween 20, Carl Roth, Germany) in PBS, and again twice in PBS for 10 min. Last 4′,6‐diamidino‐2‐phenylindole (DAPI, Thermo Fisher Scientific, United States) was added for 10 min and rinsed twice afterward. Microscopy was performed using a Thunder Imagers DMi8 (Leica Microsystems, Germany). Excitation was conducted at a wavelength of 555 nm using a filter at 630 nm. An excitation intensity of 70% and an exposure time of 40 ms were applied. A 63x oil immersion objective (HC PL APO 63x/1.40‐0.60 OIL, Leica, Germany) was used.

### 5.3 qPCR

RNA was isolated from naïve neutrophils or neutrophils incubated with LPS (50 ng/mL, Sigma‐Aldrich, United States) for 2 h at 37°C, 5 % CO_2_. The Monarch Total RNA Miniprep Kit (New England Biolabs, United States) was used following the manufacturer's instructions for use with leukocytes. The RNA was eluted in 31 µL water, and the RNA concentration was determined using a DS‐11 spectrophotometer (DeNovix, United States). The isolated RNA was stored at −70°C until further use. RNA was converted into complementary DNA (cDNA) by reverse transcription using the SuperScript IV First‐Strand Synthesis System (Invitrogen, United States) and stored at −20°C until further use. qPCR of synthesized cDNA was performed using the 5x HOT FIREPol EvaGreen qPCR Mix Plus (ROX) (Solis BioDyne, Estonia). All primers (Table S2) were generated by Microsynth AG (Switzerland) and diluted to 8 µm with nuclease‐free water.

For each sample, 4 µL 5x HOT FIREPol EvaGreen qPCR Mix Plus (ROX), 0.5 µL of both the forward and backward primers, and 10 µL of nuclease‐free water were added to 5 µL of cDNA in the qPCR plate. The samples were mixed by pipetting and centrifuged shortly for one minute at 1000 rpm. The qPCR reaction was run and measured on a qTOWER 2.2 quantitative real‐time PCR thermal cycler with qPCRsoft 3.1 software (Analytik Jena, Germany) according to the thermal cycling profile described in Table S3. After the end of the qPCR reaction, a melting curve from 60°C to 95°C was generated.

### 5.4 Catecholamine, HVA, and VMA Detection by ELISA

For cell lysis, isolated neutrophils were washed with cold PBS and then resuspended in 150 µL cold PBS. The cell suspension was tip‐sonicated with a model 120 sonic dismembrator (Fisherbrand, United States) at 70 % amplitude, 10 s on / 20 s off cycle, for 3.5 min on ice. The sample was then frozen in liquid nitrogen and thawed in an ultrasonic bath (Bransonic 221, Branson Ultrasonics, United States) three times. Cell debris was pelleted by centrifugation at 14 000 x g, 4°C, for 10 min, and the lysate was further cleaned by centrifugation through a nylon micro‐centrifuge filter (0.2 µm, Ciro, United States) at 1000 x g, 4°C, for 2 min. Neutrophils were incubated at 37°C with latrunculin A (1 µm, Tocris Bioscience, United Kingdom) for 30 min and afterward stimulated with N‐Formylmethionyl‐leucyl‐phenylalanine (fMLP, 0.5 µm, Merck, Germany) for 5 min, or with LPS (25 µg/mL, Sigma‐Aldrich, United States) or serotonin hydrochloride (100 µm, Sigma‐Aldrich, United States) for 30 min, respectively. Cells were then centrifuged at 4602 x g for 2 min, and the supernatant was treated with 1 x Halt Protease Inhibitor (Thermo Scientific, United States) while the cells were lysed for later analysis.

ELISAs for dopamine (Abcam, United Kingdom), norepinephrine (Abcam, United Kingdom), epinephrine (Abcam, United Kingdom or LDN, Germany), HVA (Abbexa, United Kingdom), and VMA (Abbexa, United Kingdom) were performed according to the manufacturer's instructions. The measurements were performed using a Hidex Sense 425‐301 (Hidex, Finland) microplate reader with a readout of ng/mL or pg/mL. The cell number per sample and sample volume were used to calculate the concentration per cell. The volume of a neutrophil was assumed as 1 pL.

### 5.5 Viability Staining

To assess viability after pre‐incubation with 1 µm latrunculin A for 30 min, 5 × 10^5^ neutrophils were stained using the Zombie NIR dye of the Zombie NIR Fixable Viability Kit (BioLegend, United States) at a dilution of 1:1000 in PBS for 15 min at room temperature in the dark. Afterward cells were washed with PBS + 1% FCS and centrifuged for 5 min at 1200 rpm and 4°C. For fixation, cells were incubated with 2% PFA on ice for 15 min, then washed again and resuspended in PBS + 1% FCS to be assessed via flow cytometry using the CytoFlex S cytometer (Beckman Coulter, United States). Data was analyzed using the Flow Jo V11.1.1 Software.

### 5.6 Fluorescence Microscopy of FFN Uptake by Neutrophils, HEK 293, HeLa, and PC‐12 Cell Lines

3 00 000 cells were seeded in a 3.5 cm cell culture dish and incubated for 30 min to ensure that they sedimented and adhered to the bottom of the dish. After sedimentation, the cells were washed once with HBSS with 2 mm calcium chloride (CaCl_2_, Carl Roth, Germany). HBSS was then added to the cells in the cell culture dish. Microscopy was performed using a Thunder Imagers DMi8 (Leica Microsystems, Germany). Excitation was performed at 475 nm without a filter with an intensity of 70 % and an exposure time of 40 ms. A 63x oil immersion objective (HC PL APO 63x/1,40‐0,60 OIL, Leica, Germany) was used. Before starting the measurement, an image was taken in bright field mode, focusing on the vesicles, which appeared as black structures. Then the measurement was started, and fluorescent false neurotransmitters 511 (FFN511, Abcam, United States) dissolved in DMSO (Sigma‐Aldrich, United States) were added simultaneously at a concentration of 2.5 µm, and the uptake was measured for 10 min. The images were obtained from Leica LAS X Thunder software and analyzed with ImageJ/Fiji.

PC12 cells (RRID: CVCL_0481) were obtained from DSMZ (Cat# ACC 159), HEK‐293 cells (RRID:CVCL_0045) were obtained from DSMZ (Cat# ACC 305) and HeLa (RRID:CVCL_0030) were also obtained from DSMZ (Cat# ACC 57). All cells were authenticated by the supplier and routinely tested for mycoplasma contamination using the MycoAlert Mycoplasma Detection Kit (Lonza), with negative results throughout the study.

Cell cultures of HeLa and HEK293 cells used in the experiments had been maintained in DMEM medium (Gibco) supplemented with 10% Fetal Bovine Serum (Gibco) and 1% penicillin/ streptomycin (Gibco). The cells were passaged every second to third day, keeping a confluency of under 70 %. Cell cultures of PC‐12 cells had been maintained in IMDM medium (Gibco) supplemented with 10% Fetal Bovine Serum (Gibco), 1% penicillin/ streptomycin (Gibco), and 1% horse serum (Gibco). All cell lines were seeded with 3 00 000 cells and used for FFN uptake experiments on the next day.

For FFN uptake in PC‐12 cells, the cells were incubated with FFN511 (Abcam, USA; 2.5 µm in DMSO) for 10 min prior to imaging using a Thunder Imager DMi8 (Leica Microsystems, Germany) using 475 nm excitation (70% intensity, 40 ms exposure) and a 63× oil immersion objective (HC PL APO 63×/1.40 OIL). Images were acquired with Leica LAS X Thunder software and analyzed in ImageJ/Fiji.

For FFN uptake experiments in HEK and HeLa cells, the cell lines were handled identically to primary neutrophils. Cells were washed with HBSS containing 2 mm CaCl_2_ and maintained in the same buffer during imaging. Microscopy was performed on a Thunder Imager DMi8 (Leica Microsystems, Germany) using 475 nm excitation (70% intensity, 40 ms exposure) and a 63× oil immersion objective (HC PL APO 63×/1.40 OIL). A bright‐field image was taken first to focus, after which FFN511 (Abcam, USA; 2.5 µm in DMSO) was added, and uptake was recorded for 10 min. Images were acquired with Leica LAS X Thunder software and analyzed in ImageJ/Fiji.

### 5.7 Primary Murine Neuron Culture and Immunofluorescence Staining

Primary ventral midbrain neurons were isolated from C57BL/6N mice. They were grown on an astrocyte feeder layer isolated from C57BL/6N cortex for 2–6 weeks before use in immunofluorescence experiments. Samples were prepared, fixed, and labeled as published previously [54].

### 5.8 Fluorescence Microscopy of FFN Exocytosis From Neutrophils

Neutrophils were adhered to glass chamber slides at 10^6^/mL concentration in HBSS (with Ca^2+^ and Mg^2+^). FFN511 was dissolved in DMSO and added to the cell suspension with a final concentration of 5 µm. Cells were incubated at 37°C, 5 % CO_2_, for 5 min, then the media was aspirated, and cells were washed 3 times with HBSS and immediately used for experiments. For FFN102 (Abcam, USA), 10 µm concentration and 30 min incubation time were used. Cells were then triggered by adding 100 µm serotonin (Thermo Fisher Scientific, United States), and the exocytosis was imaged using an Olympus IX83 microscope (Japan) coupled with a CoolLed pE‐4000 illumination system. The FFNs were excited with a 488 nm LED at 500 ms exposure time. Image sequences were acquired before, during, and after the stimulant was added with a pipette to the chamber slide. The images were obtained from Olympus cellSense software and analyzed with ImageJ.

### 5.9 Synthesis of Nanosensors

(6,5)‐chirality enriched SWCNTs (carbon ≤ 95 %, ≥ 93 % carbon as SWCNT, Signis SG65i, 0.7–0.9 nm diameter (Sigma‐Aldrich, United States) and (GT)_10_ oligonucleotide (5’‐GTGTGTGTGTGTGTGTGTGT‐3 synthesized by Sigma‐Aldrich, United States) were mixed in phosphate saline buffer (Gibco, United States) with final concentrations of 0.5 mg/mL and 50 µm, respectively. The dispersion was tip‐sonicated with a Model 120 Sonic Dismembrator (Fisherbrand, United States) at 30 % amplitude for 20 min. The nanosensor suspension was centrifuged at 16 000  × g for 30 min twice. After each centrifugation, the pellet was discarded, and the supernatant was collected for further investigation. The nanosensor suspension was stable at room temperature for several months.

### 5.10 Nanosensor Characterization

Absorption spectra of the nanosensors were collected with a UV–vis–nIR spectrometer (JASCO V‐670, Spectra Manager Software). They were diluted 1:100 in PBS to measure nIR absorbance. The concentration was then calculated by integrating the area under the curve belonging to [5, 6] SWCNTs peak and taking the extinction coefficient with an estimated length of 600 nm. For all catecholamine‐sensing experiments with neutrophils, the applied concentration was 4 nm. For acquiring the fluorescence emission spectra of the nanosensors in suspension, the concentration was 2 nm. For fluorescence spectra, the nanosensor suspension in HBSS (2 nm) was excited with a 561 nm laser coupled to an Olympus IX73 microscope at 1 s integration time. The emission spectra from 800 to 1300 nm were acquired by an Andor iDus InGaAs 491 array NIR detector attached to a Shamrock 193i spectrograph (Andor Technology Ltd., Northern Ireland).

### 5.11 Fluorescence Microscopy of Neutrophils Adhered on Nanosensors

Nanosensors were immobilized on glass surfaces by incubating the surface with 4 nm nanosensors suspension overnight at 4°C. Before measurements, the surfaces were washed with HBSS three times, and HBSS (with 2 mM Ca^2+^ and Mg^2+^) was added to the surface. Neutrophils at a concentration of 10^6^/mL in HBSS (with 2 mm Ca^2+^ and Mg^2+^) were seeded onto the nanosensor‐immobilized glass‐bottom petri dishes or slides. The neutrophils were allowed to adhere for 5 min, and the medium was aspirated to remove loosely adhered cells. Fresh medium was added to the cells, and image acquisition was started. After typically 10 s, the stimulant was added to the media while image acquisition continued. The concentration of the stimulants was 100 nm fMLP, 25 µg/mL LPS or 100 µm 5‐HT.

For imaging, we employed an Olympus BX53 or IX73 microscope, a 561 nm laser (Cobolt Jive laser, Cobolt AB, Sweden), and two cameras. An Andor Zyla 5.5 sCMOS camera, (Andor Technology Ltd., UK) for visible fluorescence and NIR InGaAs (Cheetah‐640‐TE‐1, Xenics, Belgium) camera for nIR fluorescence. Images were obtained with a 100x objective lens (UPLSAPO100XS, Olympus, Japan) and recorded with down to 100 ms exposure time and up to 15 frames per second. For visible fluorescence, a xCite 120Q fluorescence lamp and an EGFP excitation filter were used. The emission was filtered by a 650 nm short‐pass filter, a 561 nm notch filter, and a 525/50 nm bandpass filter. For nIR fluorescence the 561 nm laser was used typically at 100 mW power. Images were analyzed with ImageJ. The drift was corrected by subtracting a linear line.

### 5.12 Calcium Flux Measurement

Neutrophils at 10^6^/mL concentration in RPMI 1640 (supplemented with 10 % FBS) were incubated with Fluo‐4, AM (1 µm, Thermo Fisher Scientific, United States) at room temperature for 40 min. The cells were washed once and resuspended in HBSS (with Ca^2+^ and Mg^2+^). The cell suspension was added to glass‐bottom, black 96‐well plates. A CLARIOstar Plus plate reader was employed. Wells were scanned with (excitation 480–14 nm/emission 530‐30) 691 times at 0.41 s intervals. At *t* = 50 s and *t* = 250 s, an autoinjector pumped 10 µL of stimulant with 430 µL/s speed into the well.

### 5.13 CA Release, Diffusion, and Sensor Response Simulation

A numerical simulation was performed (in 2D) to model the release of CA (with the diffusion constant of dopamine/DA), the subsequent diffusion, and the binding dynamics of the DA molecules to nanosensors. The simulation can be divided into two main processes: A simulation of the release and diffusion of DA, and a simulation of the binding of DA molecules to the (nano)sensors based on the spatiotemporal concentration profile. Both processes were simulated with numerical methods to ensure accuracy and efficiency:

A numerical simulation of the diffusion equation (Fick's second law) was chosen to model the diffusion. For this purpose, a Runge‐Kutta method was applied, which is particularly suitable for approximating time‐dependent partial differential equations such as the diffusion equation. To describe the experimental situation, a two‐dimensional geometry of the diffusion was assumed. The structure of substrate, sensors, and cells within a thin liquid layer only allows diffusion in two dimensions. The diffusion area was divided into pixels, for each pixel, and for each time step, the dopamine concentration was calculated based on the concentration of the surrounding pixels in the previous time step. The size of the time steps was chosen sufficiently small to ensure high accuracy and stability of the simulation.

To test the reliability of the simulation, a simple geometry was considered after an instantaneous release from a point source, for which the diffusion equation can also be solved analytically in two dimensions (Figure S11A). A comparison of the simulation and the analytical solution shows only minimal deviations. The simulation only deviates from the analytical solution due to the selected boundary conditions for very long diffusion times and low concentrations that are no longer relevant for sensory analysis.

The release of DA can be incorporated into the simulation in various ways. A release at different times and at different locations is possible, but also a single release event. The release can either be simulated as coming from a point‐like source or evenly distributed over the complete surface of a cell.

The binding process was modeled probabilistically, accounting for the stochastic nature of molecular interactions. Binding events are governed by rate constants for binding and unbinding reactions. At each time step, the simulation checks each sensor for potential binding and unbinding events based on the local concentration of molecules and the number of available binding sites. This method ensures a realistic representation of the dynamic equilibrium between free and bound molecules. Every binding or unbinding event changes the local concentration in the area of the sensor. Since only one binding or unbinding event is considered per binding site and time step, the size of the time steps had to be chosen sufficiently small so that the probability of multiple binding and unbinding events at a single binding site can be neglected.

For the simulation, a series of parameters had to be tailored to fit the biological conditions, as given in Tables S4 and S5. Several runs of the simulation with different changes in the parameters were performed. Each run of the simulation leads to DA concentration mappings and bound DA molecule number mappings for each simulated time step. For analysis, two central outputs were evaluated for each run of the simulation: The number of free DA molecules in the area of the cell (a circle with radius 10 µm in the center of the simulated area) and the number of sensor‐bound DA molecules in the same area. As the bound DA molecules on the SWCNT are the cause of the fluorescence increase, we can compare the simulated number of bound DA molecules with the experimental results of the fluorescence intensity.

Out of all the five considered runs of the simulation, run 1 fits the experimental results (Figure 3D) qualitatively best. The number of bound DA molecules increases over the course of ∼30 s, before the increase decelerates and binding and unbinding come close to reaching an equilibrium. After the last release event, an unbinding due to the sensor kinetics given by k_off_ can be observed.

After investigating other simulated parameters, a scenario of a release separated into several release events over the course of several 10 s to 1 min combined with sensor kinetics described by an on rate in the order of 10^6^ M^−1^s^−1^ and an off rate in the order of 0.1 s^−1^ describes the observed results best.

The observed slow increase in the fluorescence signal cannot be caused by a single release event at one time (run 2, run 4). Due to the diffusion, already a few seconds after the release event, the DA concentration would have decreased below the detection level of the nanosensors. A mathematically possible exception to this is the case where diffusion is reduced by several orders of magnitude compared to the literature values [55], and the on‐rate is also reduced by several orders of magnitude (run 5). In this case, however, the ratio between on rate and off rate would no longer agree with the K_d_ values of the DA nanosensors known from literature [24, 28], and this option can be rejected.

Compared to even slower off‐rates (run 3), the parameters chosen in run 1 fit the experimental results better, considering the slower increase after ∼30 s and the speed of the signal decrease after the last release event. Additionally, the ratio between on rate and off rate fits best with the *K*_d_ values of the nanosensors known from literature [24, 28].

### 5.14 Isolation of Human Platelets From Whole Blood

Platelets were isolated from whole blood of healthy donors. This study was approved by the Ethics Committee Westfalen‐Lippe (approval number 2021‐657‐f‐S). Before donating blood, fully informed consent of each donor was obtained.

Whole blood was collected into citrate 9NC blood collection tubes (Sarstedt, Germany) and centrifuged at 259 x g, for 30 min. Platelet‐rich plasma (PRP) was separated and centrifuged at 259 x g, for 10 min. PGE1 at a final concentration of 6.6 µm was added to the PRP and centrifuged at 10 000 x g for 30 s. The pelleted platelets were carefully resuspended in Tyrode's solution (pH 6.2) supplemented with PGE1 at a final concentration of 5 µm. Then, the cell suspension was centrifuged again at 10 000 x g for 30 s and resuspended in Tyrode's solution (pH 6.2) with PGE1 as described before. Finally, the platelets were centrifuged at 10 000 x g for 15 s, resuspended in Tyrode's solution (pH 7.4), and kept at 37°C before starting experiments.

## 6 5‐HT Receptor Inhibition

Nanosensors were immobilized on glass surfaces by incubating the surface with 4 nm nanosensor suspension overnight at 4°C. Neutrophils at 10^6^/mL concentration in RMPI 1640 were seeded onto the nanosensor‐coated glass surface and incubated with or without 100 µm ketanserin (+)‐tartrate (ketanserin, Sigma‐Aldrich United States) and subsequently washed with buffer. Stimulation with either 100 µm 5‐HT or 1 µm 2‐[(3‐chlorophenyl)methoxy]‐6‐(1‐piperazinyl)‐pyrazine (CP809, Tocris Bioscience, United Kingdom) was performed 10 s after the start of imaging. Images were obtained with a 100x objective lens (UPLSAPO100XS, Olympus, Japan) and recorded with 100 ms exposure time and at 5 frames per second. For nIR fluorescence imaging the 561 nm laser was used at 100 mW power. Images were analyzed with ImageJ. Drift was corrected by subtracting a linear line.

### 6.1 Neutrophil‐Platelet Interaction

Nanosensors were immobilized on glass surfaces by incubating the surface with 4 nm nanosensors suspension overnight at 4°C. For control measurements, thrombin (1 Unit/mL, Sigma‐Aldrich, United States) was added onto blank nanosensors or onto nanosensors with adhered platelets. For neutrophil measurements, neutrophils at 10^6^/mL in HBSS were seeded on a nanosensor‐coated glass surface and incubated for 5 min before platelets were added and incubated for 5 min. The measurement was started, and thrombin (1 Unit/mL) was added after 10 s. Images were obtained with a 100x objective lens (UPLSAPO100XS, Olympus, Japan) and recorded with 200 ms exposure time and at five frames per second rate. For nIR fluorescence 561 nm laser was used at 120 mW power. Images were analyzed with ImageJ. The drift was corrected by subtracting a linear line.

For serotonin receptor inhibition, neutrophils and platelets were seeded onto the nanosensors and treated with 100 µm ketanserin for 10 min before stimulation. The cells were rinsed with HBSS. The measurement was started, and thrombin (0,01 Unit/mL) was added after 10 s. Images were obtained with a 100x objective lens (UPLSAPO100XS, Olympus, Japan) and recorded with 200 ms exposure time and at five frames per second rate. For nIR fluorescence, a 561 nm laser was used at 120 mW power. Images were analyzed with ImageJ.

### 6.2 NETosis Assay

Neutrophils were seeded on glass‐bottom 96‐well plates (10 000 per well), activated with PMA (100 nm, Sigma‐Aldrich, United States), and simultaneously incubated with dopamine hydrochloride, DL‐norepinephrine hydrochloride, (±)‐epinephrine hydrochloride or serotonin hydrochloride (all Sigma‐Aldrich, United States) at 37°C, 5 % CO_2_. After a 3 h incubation time, the cells were fixed with 2 % PFA to stop NET formation and stored overnight at 4°C. Cells were then washed once, and the chromatin was stained with Hoechst 33342 at room temperature. Neutrophils were imaged with an Eclipse Ti2 microscope (Nikon Instruments) equipped with an Orca Fusion BT camera (Hamamatsu Photonics) or an Axiovert 200 microscope (Zeiss) with a CoolSNAP ES camera (Photometrics). Eight images from random regions were collected for each well. For all experiments, the number of decondensed nuclei and the total cell count were quantified with ImageJ.

### 6.3 Aggregometry

Aggregometry experiments were performed using an eight‐channel aggregometer (PAP‐8E, möLab, Germany). Before the start of the experiment, MgCl_2_ and CaCl_2_ (both Sigma‐Aldrich, United States) were added to the platelets to achieve a final concentration of 1 and 2 mm, respectively. The platelets were incubated with the catecholamines (dopamine hydrochloride, DL‐norepinephrine hydrochloride, (±)‐epinephrine hydrochloride, all Sigma‐Aldrich, United States) for approximately 2 min before stimulation with thrombin (1.2 U/mL, Sigma‐Aldrich, United States), and the platelet response was measured throughout. The measurements were continued for 30 min or until the aggregation had reached a plateau. The magnitude of aggregation was determined by finding the maximum aggregation throughout the measurement. The aggregation speed was determined by performing a Michaelis‐Menten least squares curve fit of the aggregation data generated in the first 20 min after the stimulation of aggregation and is presented as the *K*_m_ value.

### 6.4 Transcriptomics

Human transcriptome data were obtained from the GSE35590 dataset generated using the Affymetrix Human Exon 1.0 ST Array platform. The raw CEL files were processed in R utilizing the oligo package (version 1.68.2) to perform background correction, quantile normalization, and summarization through the Robust Multi‐array Average (RMA) method. The resulting expression matrix was extracted using the Biobase package (version 2.64.0) and subsequently exported for downstream analysis in Python.

Probe annotation was conducted based on the manufacturer‐provided annotation file (HuEx‐1_0‐st‐v2.na36.hg19.transcript.csv). In cases where multiple probe sets mapped to the same gene symbol, expression values were averaged to generate a single representative value per gene. To maintain biological specificity, the analysis was restricted to genes included in a manually curated list focusing on monoaminergic and inflammatory pathways, with all other genes excluded from further consideration.

To account for baseline gene expression differences and highlight temporal dynamics, expression values were z‐score normalized across time points for each gene individually. Genes exhibiting missing or non‐finite values across all time points were systematically removed from the dataset. The processed expression matrix underwent hierarchical clustering using Euclidean distance as the metric and average linkage as the clustering method.

For functional module analysis, genes were grouped into predefined categories (Table S6). For each category, mean z‐scores were calculated at every time point to summarize transcriptional activity. To capture intra‐category variability, 95% confidence intervals (CIs) were estimated by bootstrapping across all genes within each category. This strategy is conceptually related to previous approaches for summarizing gene module dynamics [64, 65, 66, 67].

In addition, an inflammation score was calculated based on the mean z‐scores of 26 predefined inflammation‐related genes (i.e. BCL2A1, C5AR1, CSF3R, CXCR1, CXCR2, ELANE, FCGR3A, FPR1, FUT4, IL1B, IL1R1, IL1R2, IL6, IRAK3, ITGAM, ITGB2, MMP9, MPO, NFKBIA, PRTN3, PTGS2, S100A12, S100A8, S100A9, TLR4, TNF) that are involved in cytokine signaling, oxidative burst, or neutrophil activation. For each time point, z‐scores were averaged across all inflammatory genes per sample. Group‐wise comparisons between baseline and later time points were assessed using Welch's t‐tests, and statistically significant differences were annotated accordingly. This analysis allowed quantification of the coordinated inflammatory response over time.

A supplementary analysis focused specifically on serotonin receptor genes, which were further annotated according to their known signaling effects (activating or inhibiting) based on UniProtKB/Swiss‐Prot annotations. These functionally distinct receptor subtypes were analyzed separately to evaluate potential differences in their transcriptional trajectories over time.

All downstream analyses and visualizations were implemented in Python (version 3.10.16, conda‐forge distribution) using standard scientific computing packages including numpy (version 1.26.4), pandas (version 2.2.3), scipy (version 1.12.0), seaborn (version 0.12.2), and matplotlib (version 3.10.1). The data processing and visualization workflow was executed within the JupyterLab environment (version 4.3.3) using ipykernel (version 6.29.5) and notebook (version 7.3.1) interfaces.

### 6.5 Statistics

Statistical tests were performed with single‐cell data when available. Data was checked for normality using Shapiro‐Wilk tests, and consequent statistical tests were chosen accordingly and are listed in the respective figure captions. One sample *t*‐test or one‐sample Wilcoxon tests were chosen for normalized data. Paired *t*‐test was used to compare two contiguous measurements. To test significance between three or more independent groups, a one‐way ANOVA test was chosen. Kruskal‐Wallis test with optional Dunn's multiple comparisons test and Mann‐Whitney U test were chosen for non‐parametric data. Tukey's post hoc test was used for transcriptome data. Data are expressed as mean ± SEM, mean ± SD for epinephrine ELISA experiments or mean ± 95% confidence intervals for transcriptome data. Statistical analyses were performed using Origin and GraphPad Prism 9.5.1. Values with p < 0.05 were considered significant: *p < 0.05. **p < 0.01. ***p < 0.001. For a full overview of *n* numbers (independent donors) and *N* numbers (individual cells), see Table S9.

### 6.6 Graphics

The TOC figure (Erpenbeck, L. (2026) https://BioRender.com/4tl6sdw) and other graphics were created with BioRender and partially altered with Inkscape (Erpenbeck, L. (2026) https://BioRender.com/zuk80j3 and Erpenbeck, L. (2026) https://BioRender.com/30qtaja).

## Author Contributions

J.M., A.S., M.D., and F.W. shared first co authors. S.K. and L.E. conceived the project. J.M., A.S., M.D., S.J., I.B., G.W., S.K., and L.E. developed the methodology. J.M., A.S., M.D., F.W., B.H., J.G., M.S., S.J., S.S., M.W., M.B., M.S., E.N., G.W., S.K., and L.E. performed the investigation. J.M., A.S., M.D., G.W., and F.W. were responsible for data visualization. S.K. and L.E. acquired funding, administered the project, and supervised the research. M.D., M.S., A.S., J.M., S.K., and L.E. wrote the original draft of the manuscript. J.M., A.S., S.K., L.E., E.N., and I.B. reviewed and edited the manuscript.

## Funding

Deutsche Forschungsgemeinschaft (DFG, German Research Foundation) under Germany´s Excellence Strategy – EXC 2033 – 390677874 – RESOLV (S.K.)“Center for Solvation Science ZEMOS” funded by the German Federal Ministry of Education and Research BMBF and by the Ministry of Culture and Research of Nord Rhine‐Westphalia (S.K.). This work was funded by the DFG (L.E., S.K.). LE was supported by the TRR332. I.B. acknowledges funding from the DFG (BO 3643/10‐1). L.E. received funding from the IZKF Münster (Erp2/016/23).

## Ethics Statement

This study was approved by the Ethics Committee Westfalen‐Lippe (approval number 2021‐657‐f‐S). Before donating blood, fully informed consent of each donor was obtained.

## Conflicts of Interest

SK is listed on patent applications about nanosensor technology used in this work.

## Supporting information




**Supporting File**: advs75835‐sup‐0001‐SuppMat.pdf.

## Data Availability

All data are available in the main text or the supplementary materials. Raw data will be uploaded to a repository once the paper has been accepted.
