# Supplementary material for: Multimodal Imaging Reveals Rapid Catecholamine Uptake and Release by Neutrophils
Source: Adv Sci (Weinh). 2026 Jun 18;13(47):e24193. doi: 10.1002/advs.202524193 (PMC13336083; doi:10.1002/advs.202524193)
Supplement: Supplementary file 1 [file ADVS-13-e24193-s001.pdf]

Supplementary Materials for

Multimodal imaging reveals rapid catecholamine uptake and release by neutrophils

Jennifer Mohr[1]+, Anne Schmitz[2]+, Meshkat Dinarvand[3]+, Franziska Wulfert[2], Sangeetha Shankar[2], Bjoern F. Hill[1], Michael Wojak[2], Juliana Gretz[1], Marie Britz[2], Elsa Neubert[4], Magdalena Shumanska[5], Sofia Kaushik[6], Linda Kartaschew[1], Ivan Bogeski[5], James Daniel[6], Sebastian Jung[7], Johannes Eble[8], Guido Wabnitz[9], Luise Erpenbeck[2]*, Sebastian Kruss[1,3,10]*

Corresponding authors: luise.erpenbeck@ukmuenster.de, sebastian.kruss@rub.de

The PDF file includes:

Figs. S1 to S13
Tables S1 to S9

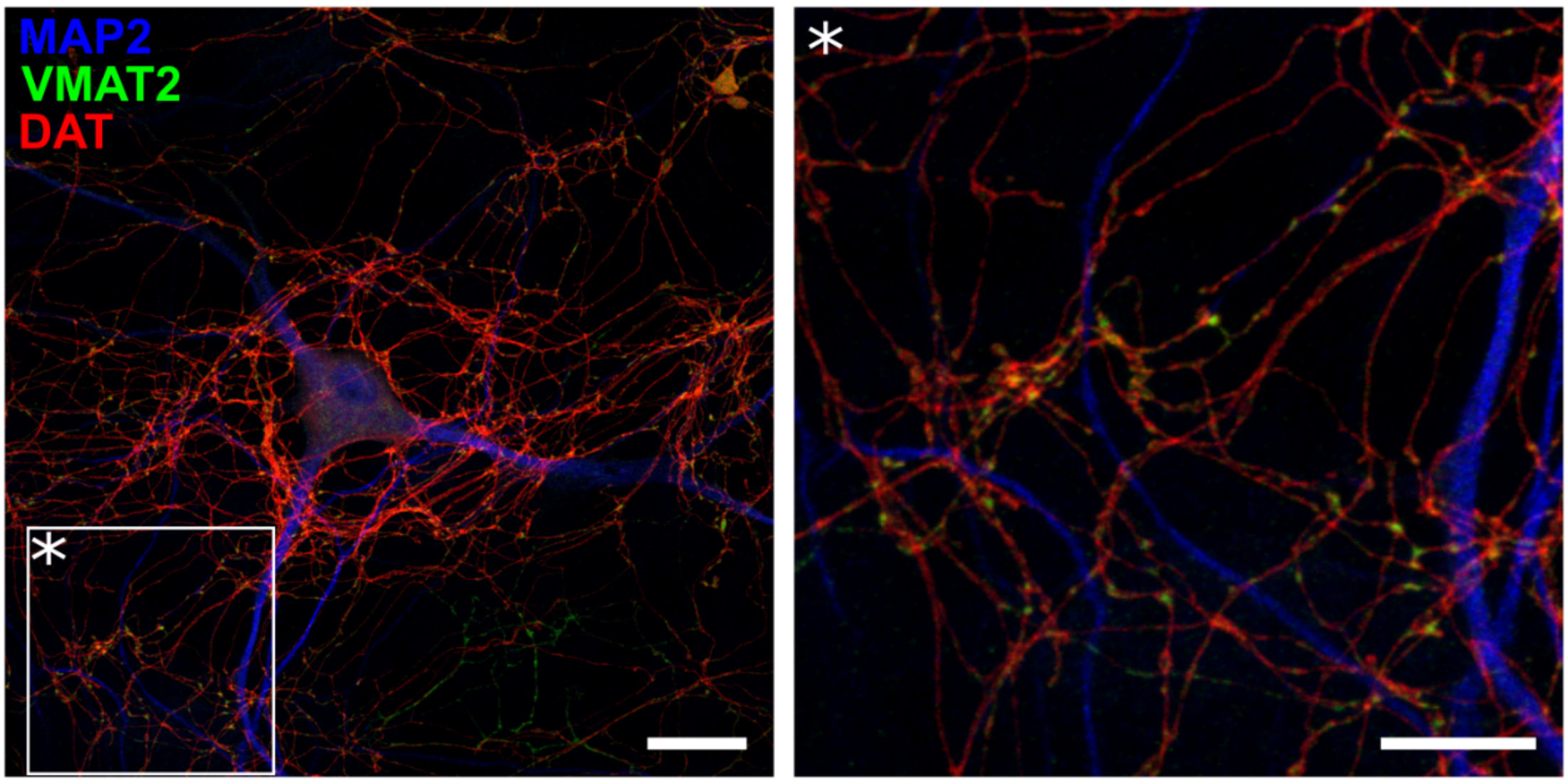

Fig. S1. *Primary mouse ventral midbrain neurons cultured on astrocytes and stained for microtubule-associated protein 2 (MAP2, blue), vesicular monoamine transporter 2 (VMAT2, green) and dopamine transporter (DAT, red). Scale bar is 20 µm in the overview (left) and 10 µm in the detailed view (right).*

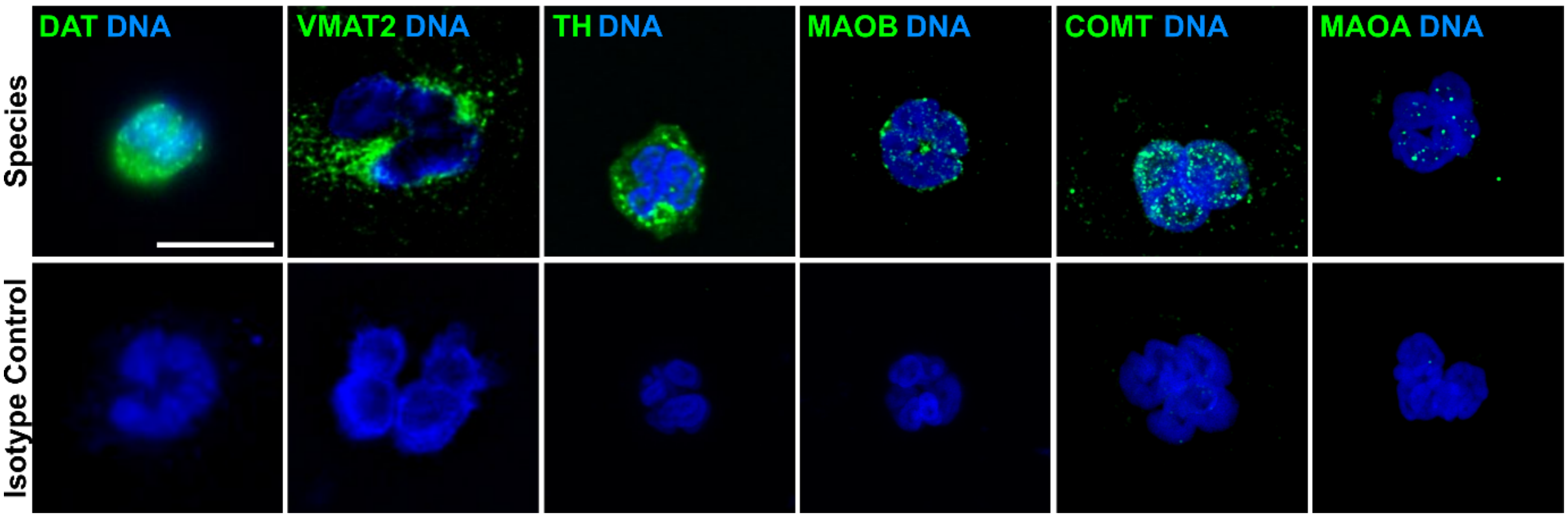

Fig. S2. *Neutrophils are stained for dopamine transporter (DAT), vesicular monoamine transporter 2 (VMAT2), tyrosine hydroxylase (TH), monoamine oxidase B (MAOB), catechol-O-methyltransferase (COMT), monoamine oxidase A (MAOA) (all green), and DNA (blue). Accordingly, the isotype controls (all rabbit IgG, green) with DNA (blue) are shown under the respective species (Each immunofluorescence and isotype staining n > 5 donors). Scale bar is 10 µm.*

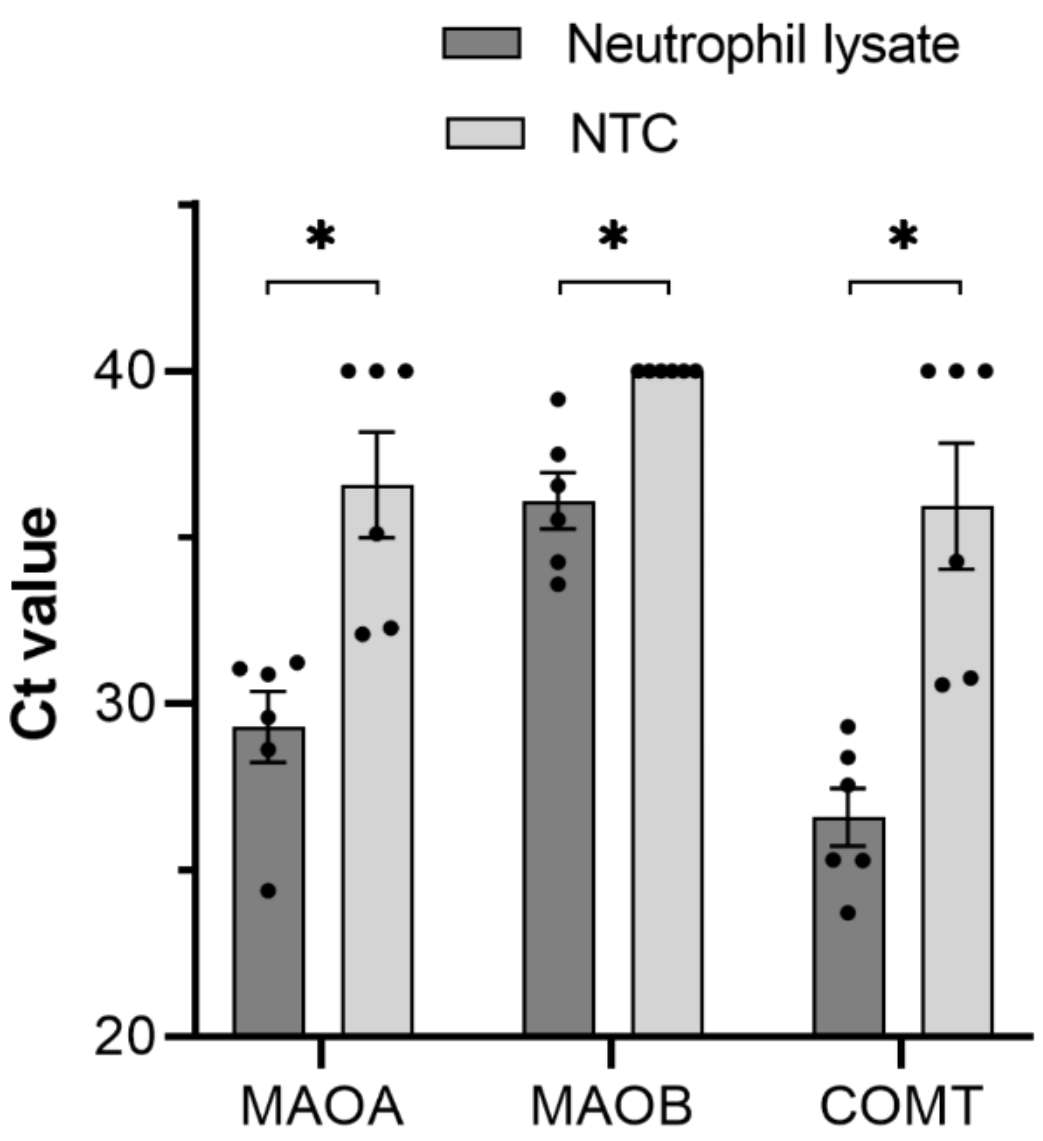

Fig. S3. *MAOA, MAOB, and COMT are expressed at the RNA level as determined by qPCR (n = 6 donors, mean ± SEM, Wilcoxon matched-pairs signed rank test, NTC: no template control).*

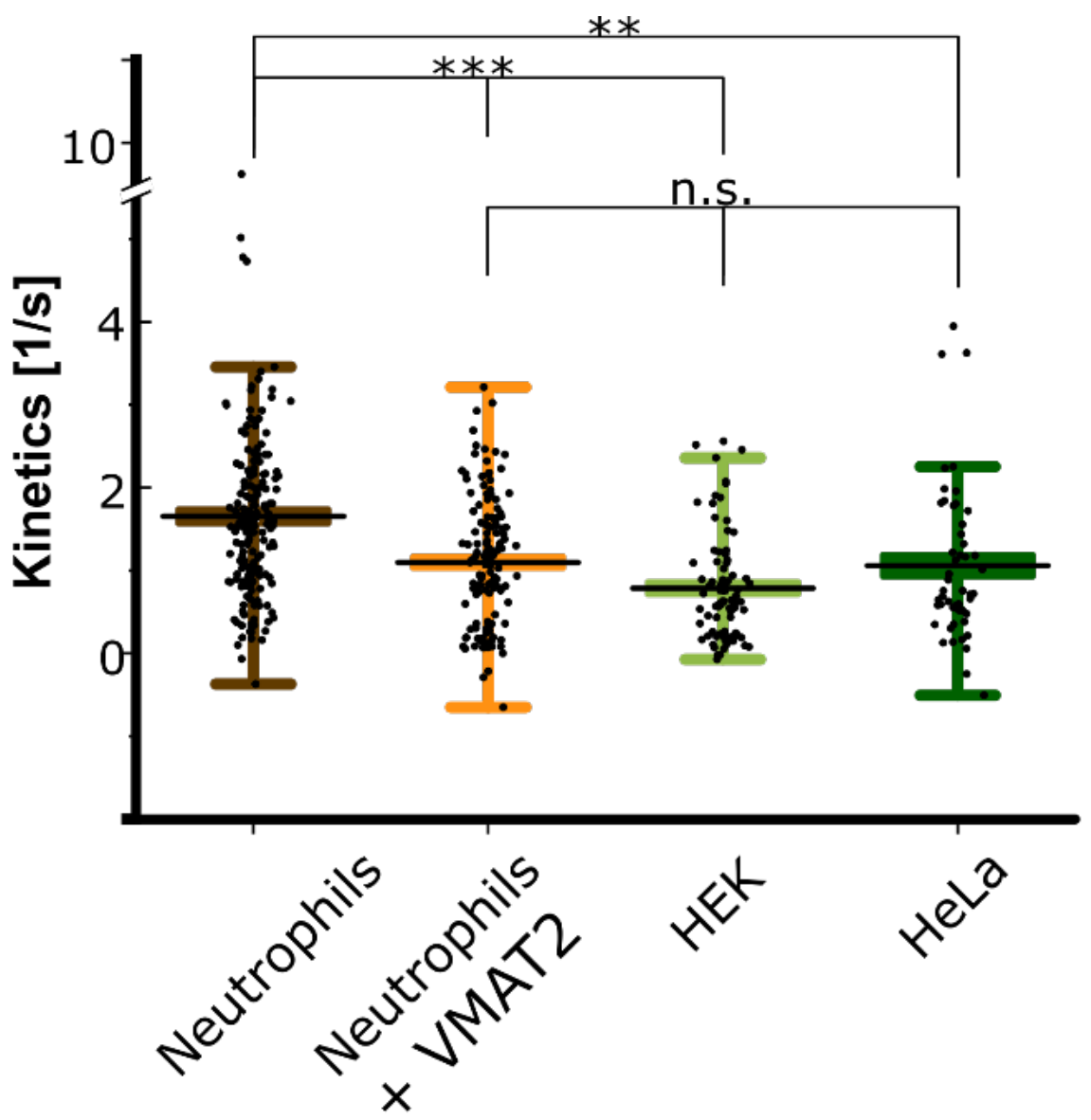

Fig. S4. *Uptake kinetics of 2.5 nM FFNs of control neutrophils, inhibited neutrophils (+100 µM VMAT2 inhibitor), HEK, and HeLa cells. VMAT2 inhibitor (Tetrabenazine) was incubated for 10 min prior the FFN addition. n = 5 donors, N ≥ 6 cells, mean ± SEM, one-way ANOVA, p-value ** < 0.01, *** < 0.001.*

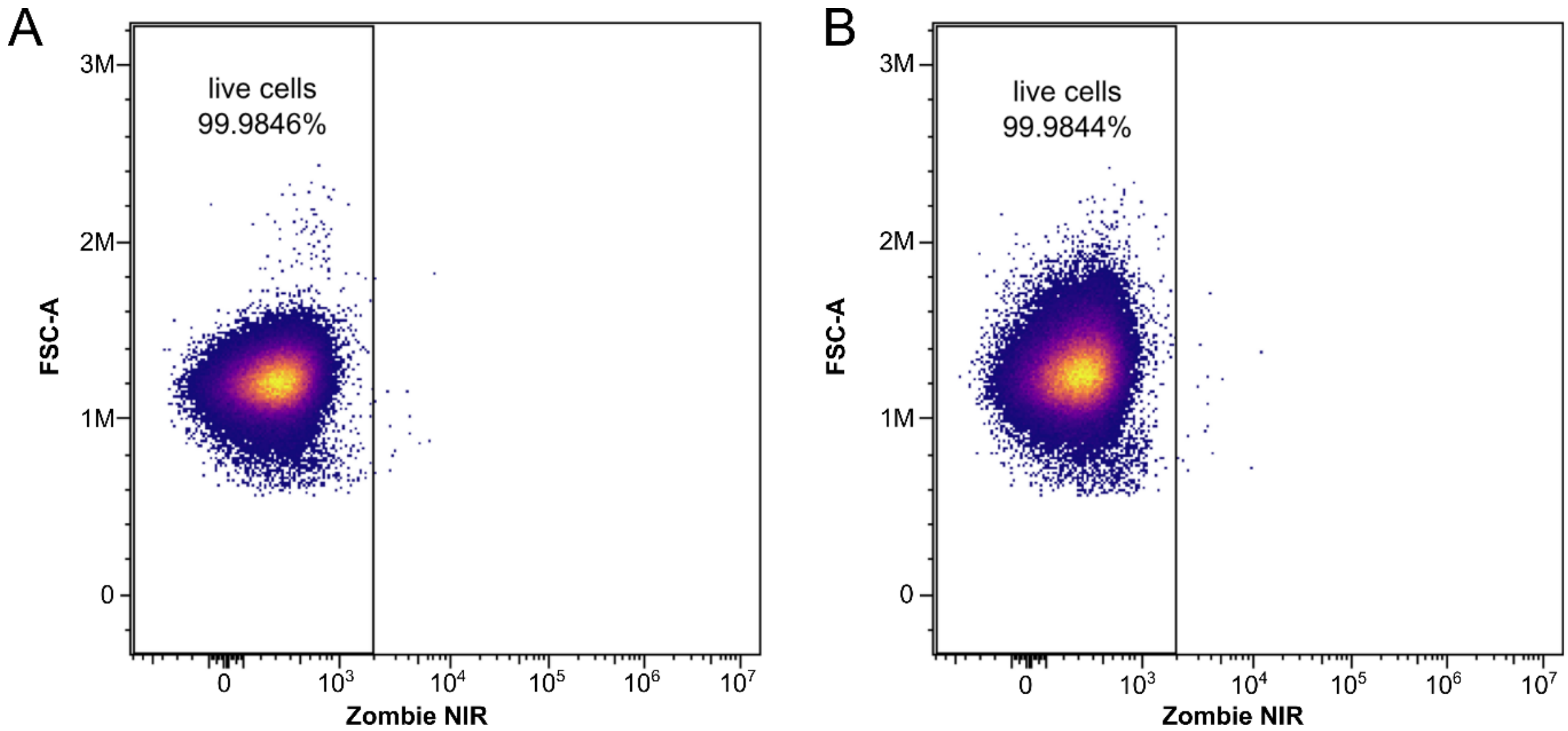

Fig. S5. *Viability staining with Zombie NIR of untreated neutrophils (left) and neutrophils incubated with 1 µM latrunculin A for 30 min (right). 1 x 10^5 events were recorded via flow cytometry and analyzed using FlowJo V11.1.1. Live cells were gated as Zombie NIR negative.*

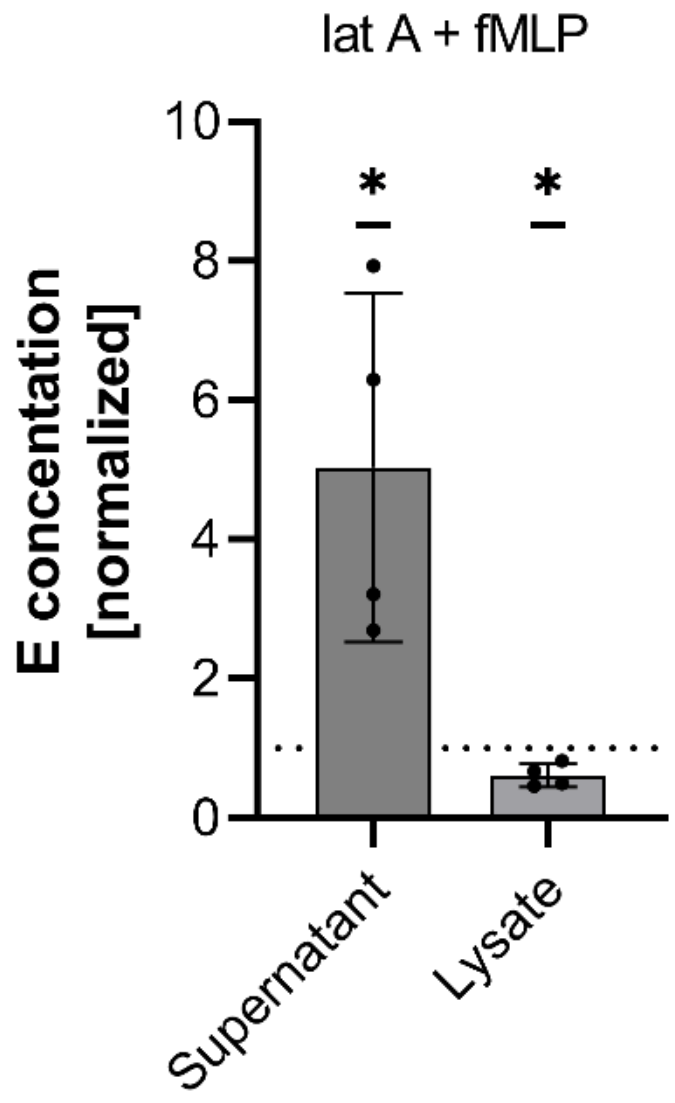

Fig. S6 *Epinephrine (E) concentration in neutrophil supernatant and lysate after pre-treatment with 1 µM latrunculin A for 30 min and stimulation with 0.5 µM fMLP for 5 min as determined by ELISA (n = 4 donors, mean ± SD, one sample t-test: p-value * < 0.05). Normalized to the control without stimulation.*

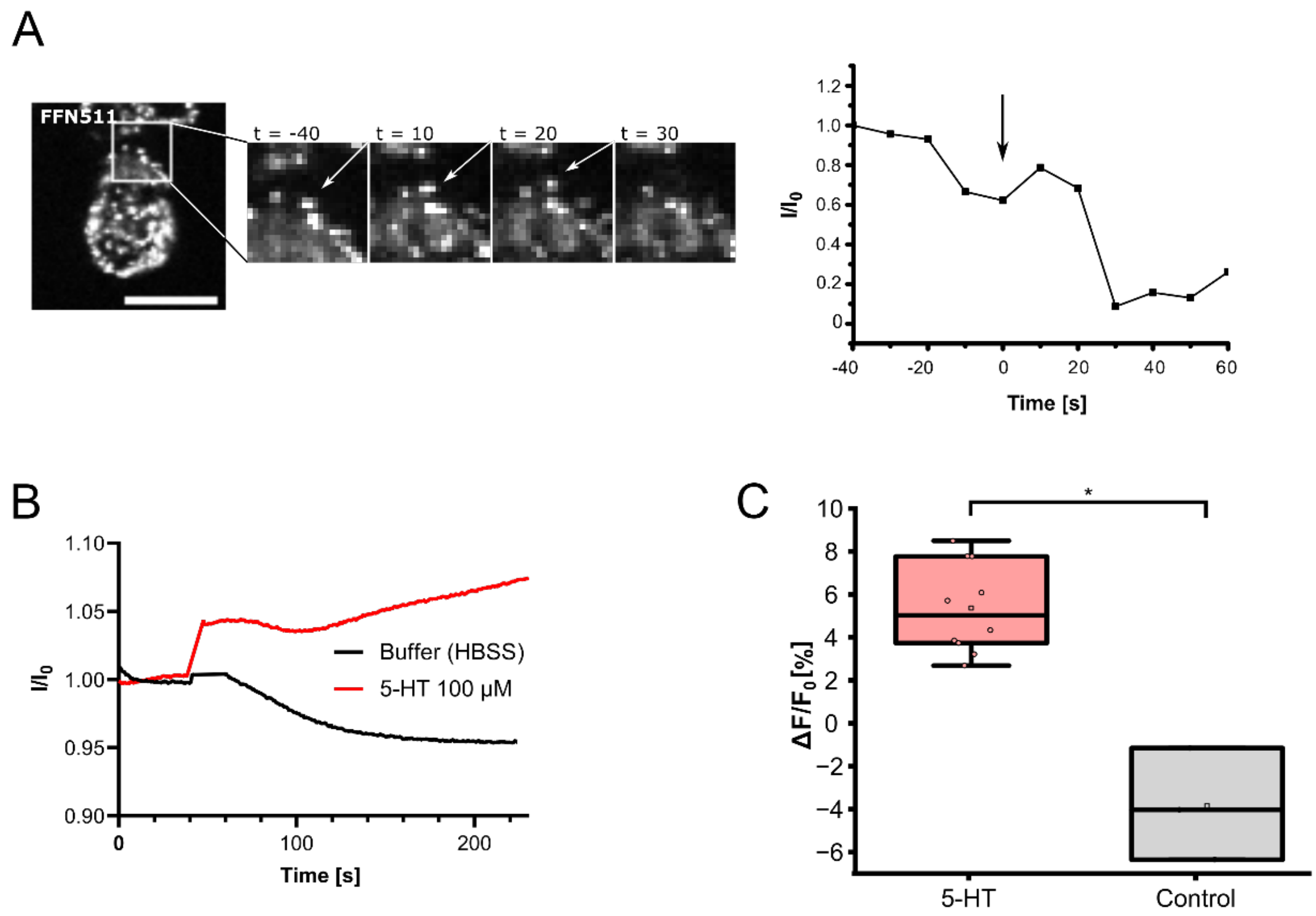

Fig. S7. *(**A**) Tracking of single FFN511 vesicles in a neutrophil before, during, and after stimulation with 100 µM serotonin, over time. Vesicle trafficking toward cell membrane, fusion with membrane, and extracellular release of FFN511 are observed and analyzed and steps in the intensity trace (right) indicate exocytosis. Scale bar is 10 µm. (**B**) Fluorescence intensity changes of the extracellular region around an exemplary cell overtime when cells are stimulated with serotonin (5-HT) compared to the control cell which is stimulated with HBSS (cell incubated with FFN102). The increase of extracellular fluorescence indicates exocytosis. (**C**) Endpoint measurements of fluorescence intensity of the extracellular region around the cells over time when cells are stimulated with serotonin (5-HT) compared to control cells stimulated with HBSS (cells incubated with FFN102) (n = 1 donor, N ≥ 3 cells, mean ± SEM, paired t-test, p-value * < 0.05).*

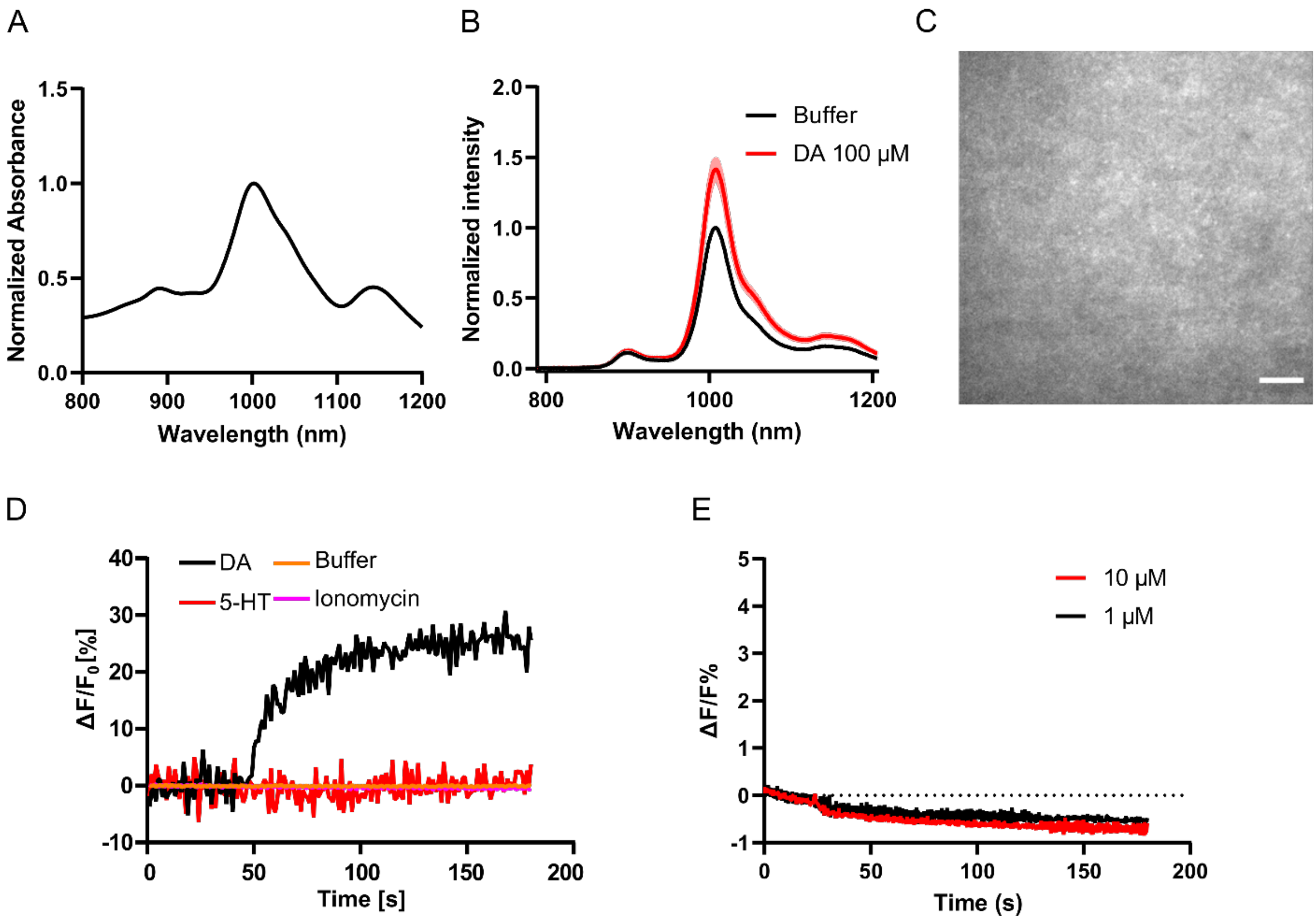

Fig. S8. *(**A**) nIR absorbance spectrum of nanosensor suspension. (**B**) Normalized fluorescence emission spectra of nanosensor in suspension before and after dopamine (DA) addition in PBS. Shades = SD (n = 3). (**C**) Nanosensor surface coverage imaged with a nIR camera. Scale bar is 10 µm. (**D**) The fluorescence response of nanosensors to dopamine, serotonin, ionomycin (blank nanosensors), and buffer (neutrophil-adhered on nanosensors) over time. (**E**) Fluorescence signal of nanosensor exposed to H_2O_2 in PBS (t = 25 s) does not increase and the decrease is most likely a z-drift of the focus.*

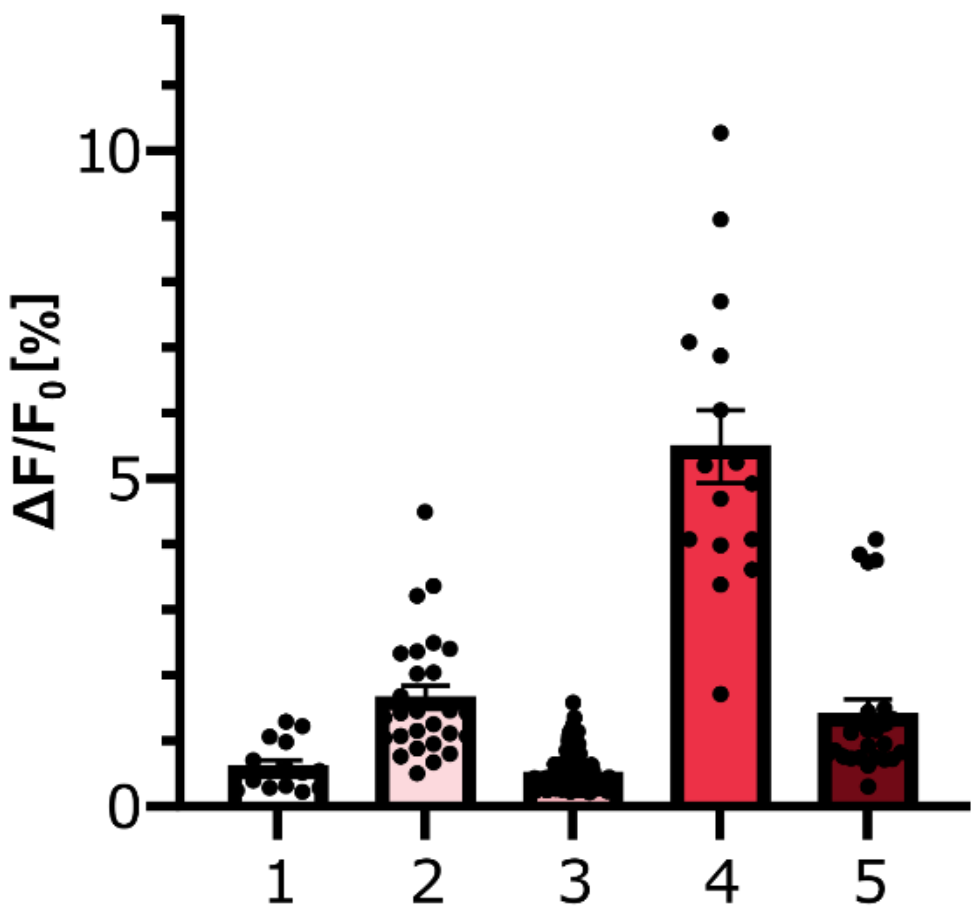

Fig. S9. *The maximum fluorescence response of nanosensors after release of CA from neutrophils stimulated by serotonin. Each bar represents an independent experiment from a different blood donor and each dot is a single neutrophil (n = 5 donors, N ≥ 15 cells, mean ± SEM). The data shows the heterogeneity between individual cells, which could also be attributed to different adherence/distance to the nanosensor layer.*

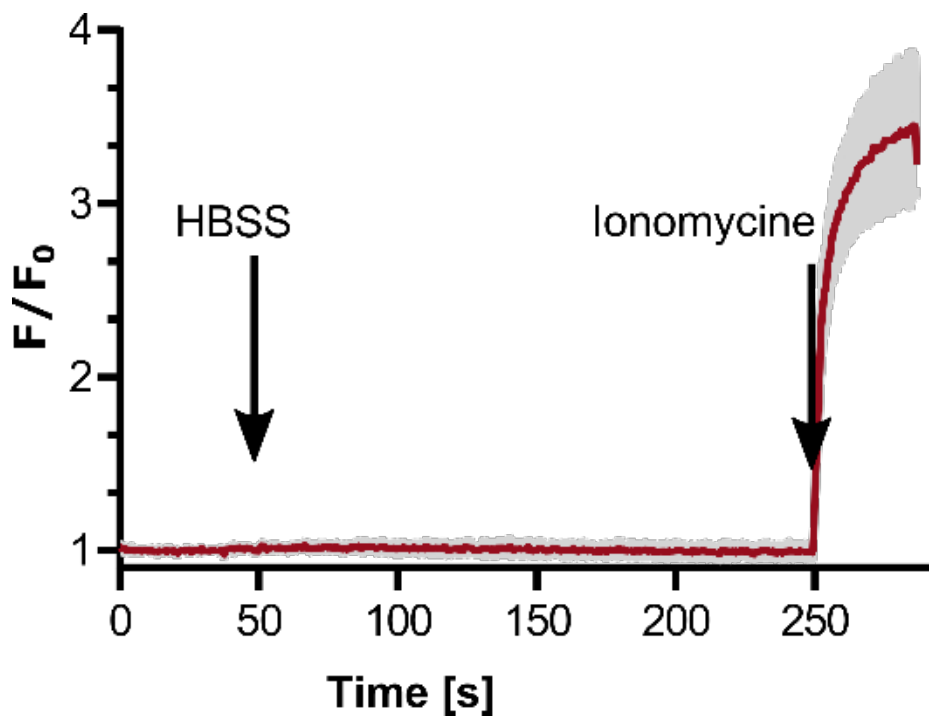

Fig. S10. *Fluorescence signal of exemplary neutrophils incubated with Fluo-4 AM, Ca^{2+} indicator, over time when HBSS (negative control) is added at t = 50 s and ionomycin (Ca^{2+} ionophore, positive control, 5 µM) is added at t = 250 s.*

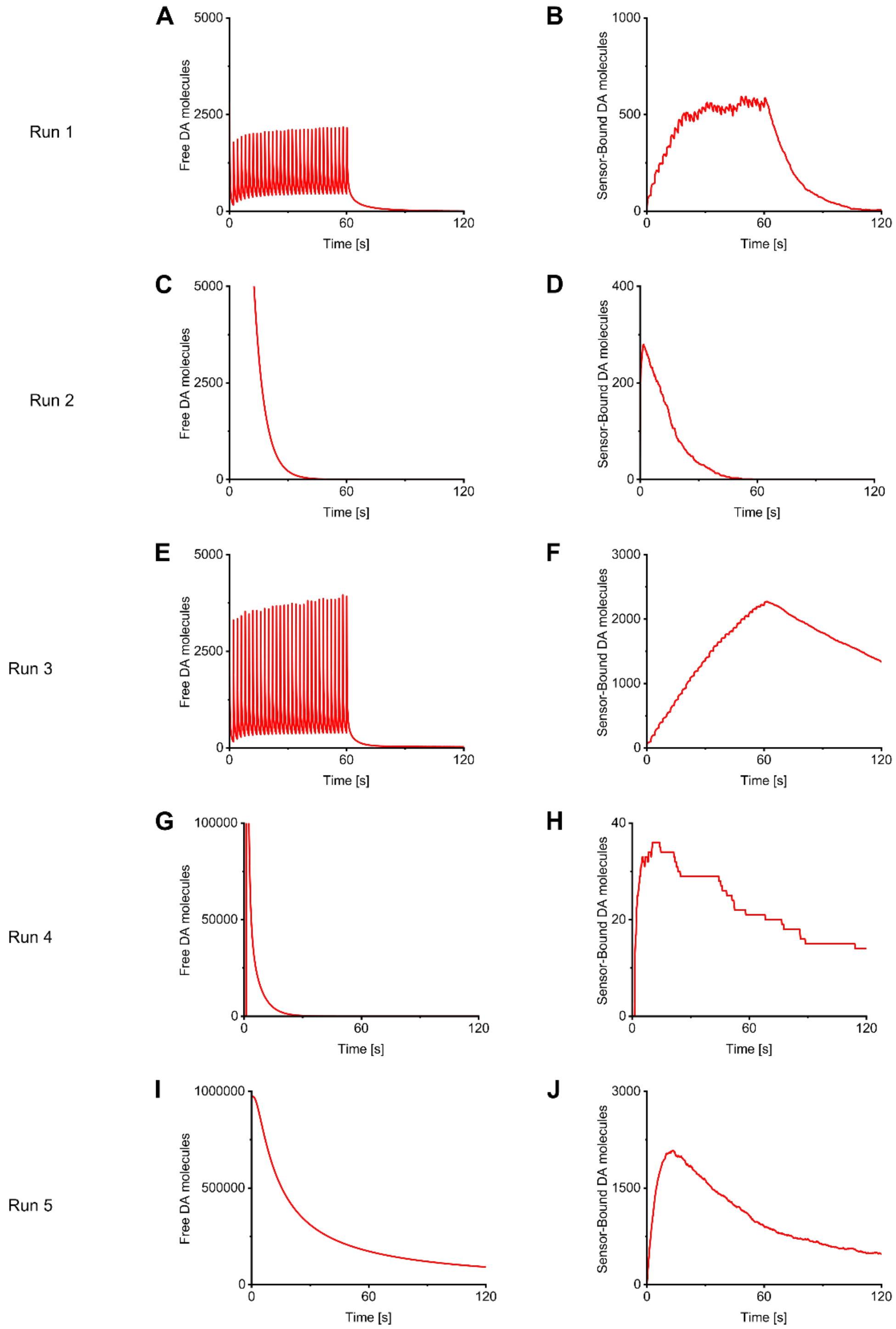

Fig. S11. *DA release, diffusion, and sensor response simulation:* ***A, C, E, G, I:*** *Free DA molecules under the area of the simulated cell in the center of the simulation area.* ***B, D, F, H, J****: Number of sensor-bound DA molecules (=sensor response) in the area of the simulated cell in the center of the simulation area. Note that only the sensor response can be measured experimentally. Data for five different preliminary runs of the simulation according to Table S5.*

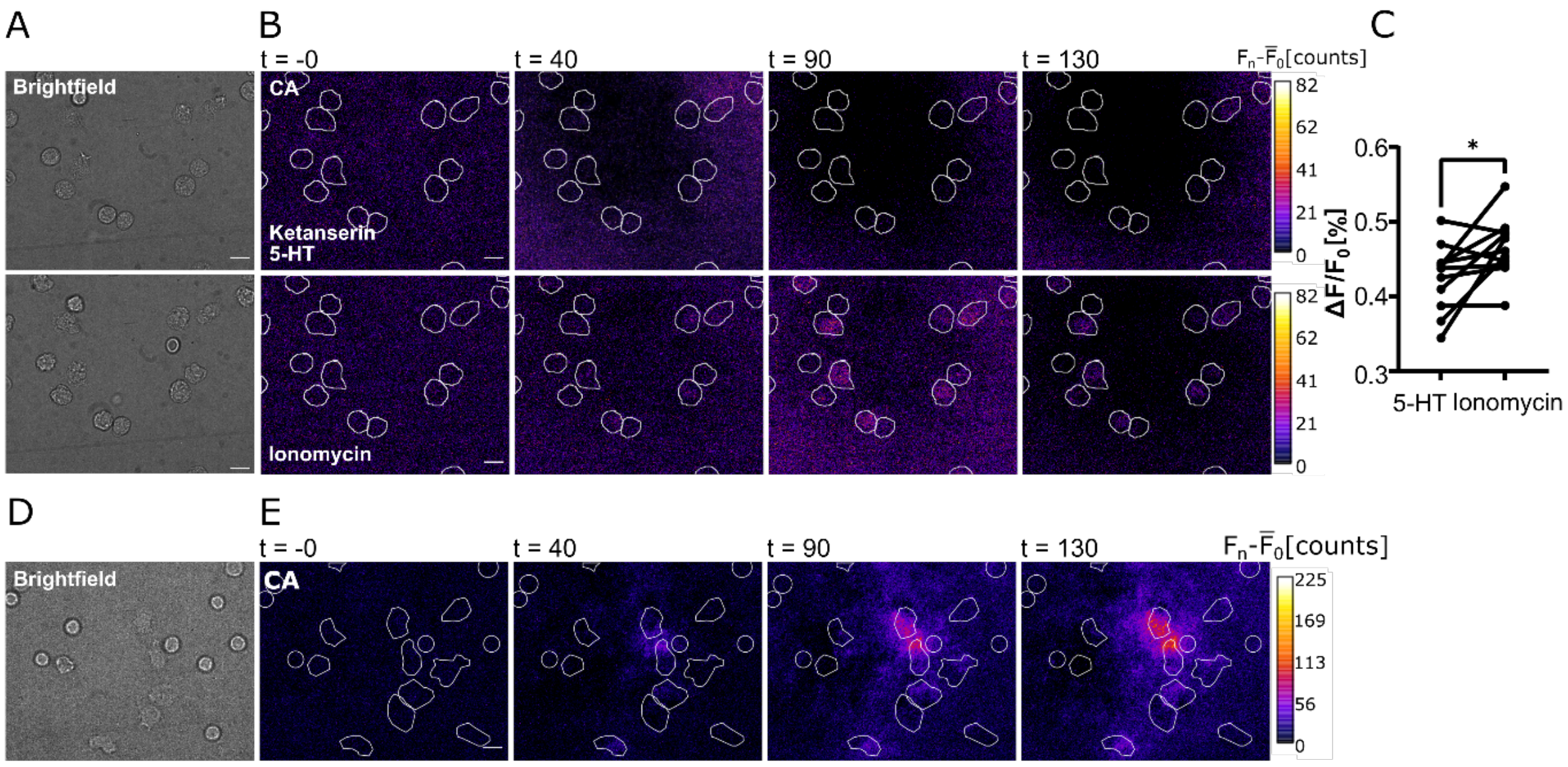

Fig. S12. ***(A)*** *Brightfield image and* ***(B)*** *nIR image sequences of neutrophils adhered on a nanosensor surface. Background is measured 10 s before addition of serotonin ($\overline{F}$). Neutrophils are first incubated with ketanserin (100 µM) and stimulated with serotonin (100 µM) at t = 0 (top). The same cells were then stimulated with 5 µM ionomycin (bottom).* ***(C)*** *Maximum fluorescence signal after stimulation of exemplary cells in section (A, B) (n =1 donor, N = 11 cells, paired t-test: p-value * < 0.05).* ***(D)*** *Brightfield image and* ***(E)*** *nIR image sequence of neutrophils adhered on nanosensor surface before and after stimulation with CP809 (1 µM). Background is measured 10 s before addition of serotonin ($\overline{F}$).*

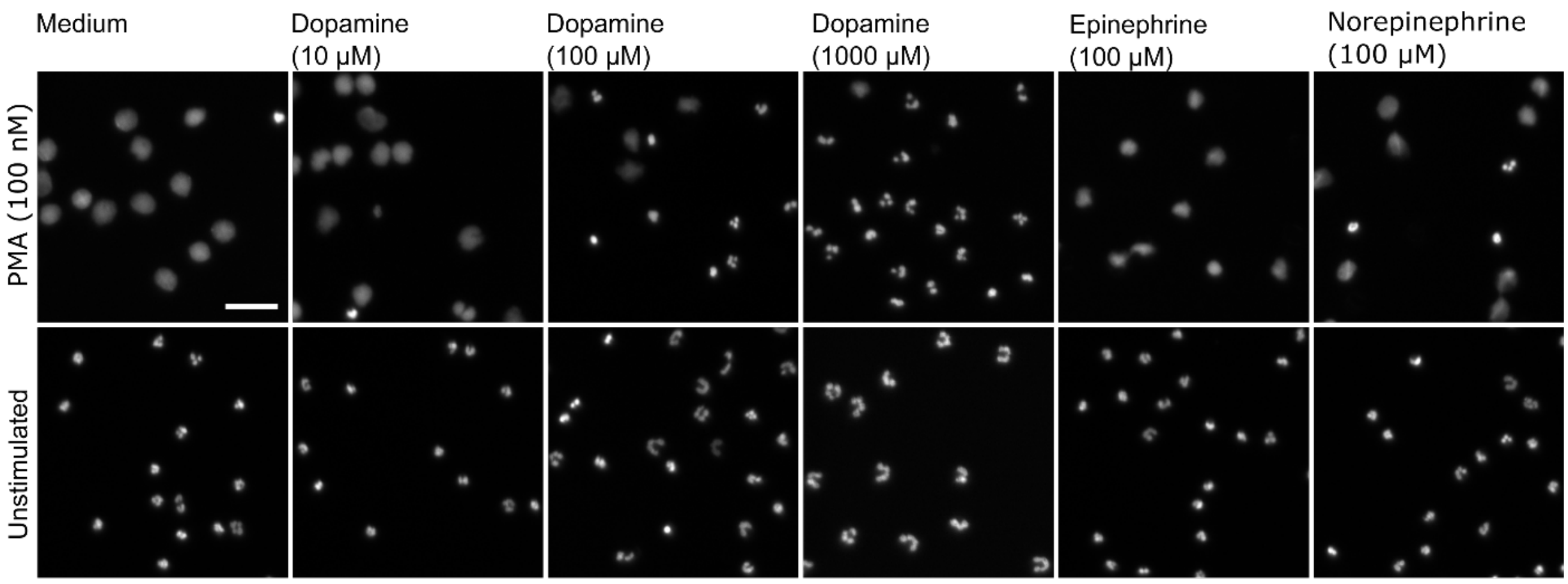

Fig. S13. *NETosis assay of neutrophils, stimulated with PMA (top) or unstimulated (bottom) and treated with different concentrations of catecholamines (10, 100 and 1000 µM dopamine, 100 µM epinephrine and 100 µM norepinephrine as indicated). The chromatin is stained with Hoechst. Images were taken after 180 min. Scale bar is 40*

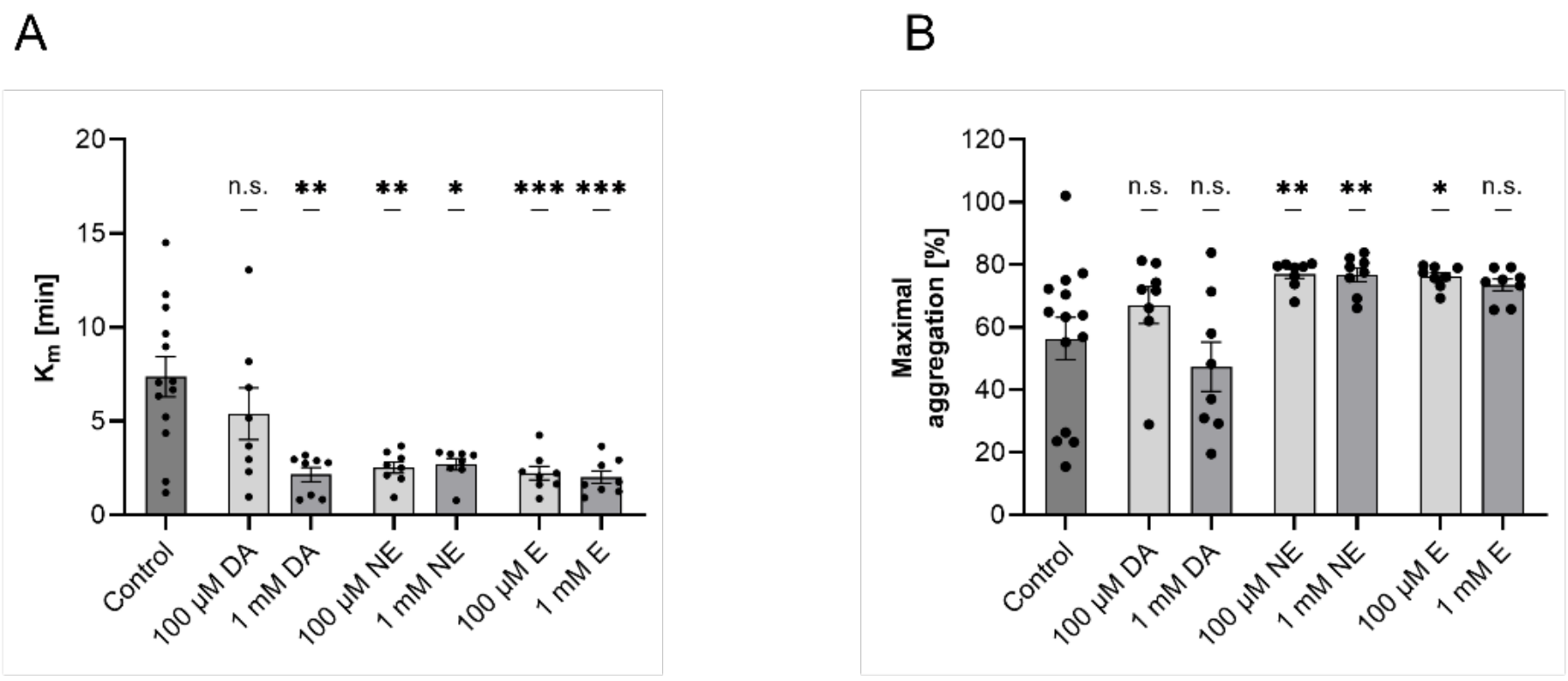

Fig. S14. (A, B*)*** *Platelets are stimulated with thrombin in combination with CAs or thrombin alone (control) to determine the aggregation speed (A) or the aggregation maximum (B) using aggregometry.* K_m *represents the time until half-maximal aggregation. (n ≥ 8, mean ± SEM, Kruskal-Wallis test with uncorrected Dunn's test: p-value * < 0.05, ** < 0.01).*

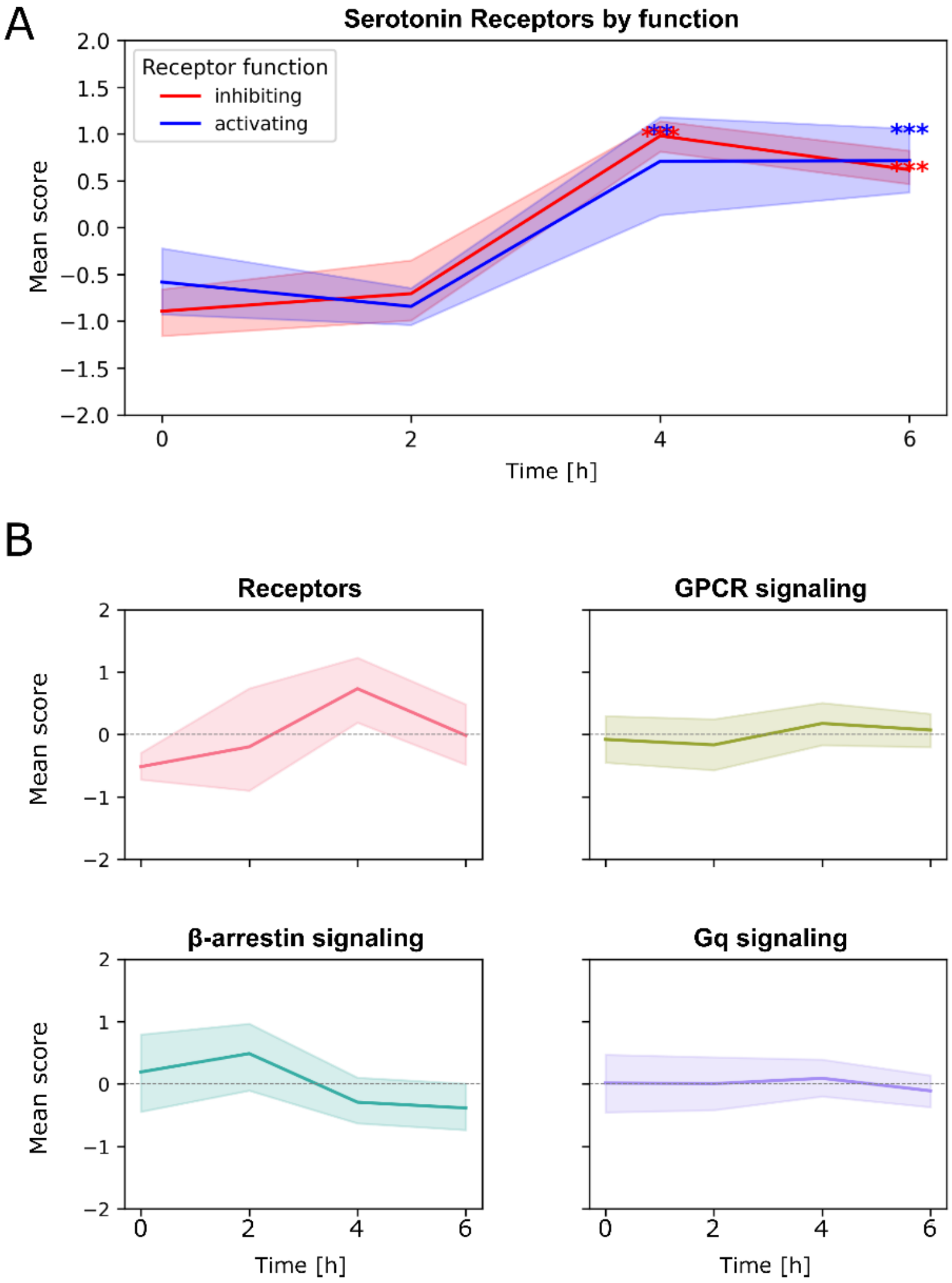

Fig. S15 *Neutrophils transcriptionally regulate monoaminergic signaling pathways during systemic inflammation. (**A**) Serotonin receptors by their annotated functional mode—activating (blue) vs. inhibiting (red). **(B)** Mean z-scored expression of gene sets grouped by functional category: receptor expression, GPCR signaling, beta-arrestin signaling and Gq signaling. (n =4 donors, mean ± 95% confidence intervals, computed from bootstrapped means across genes within each category).*

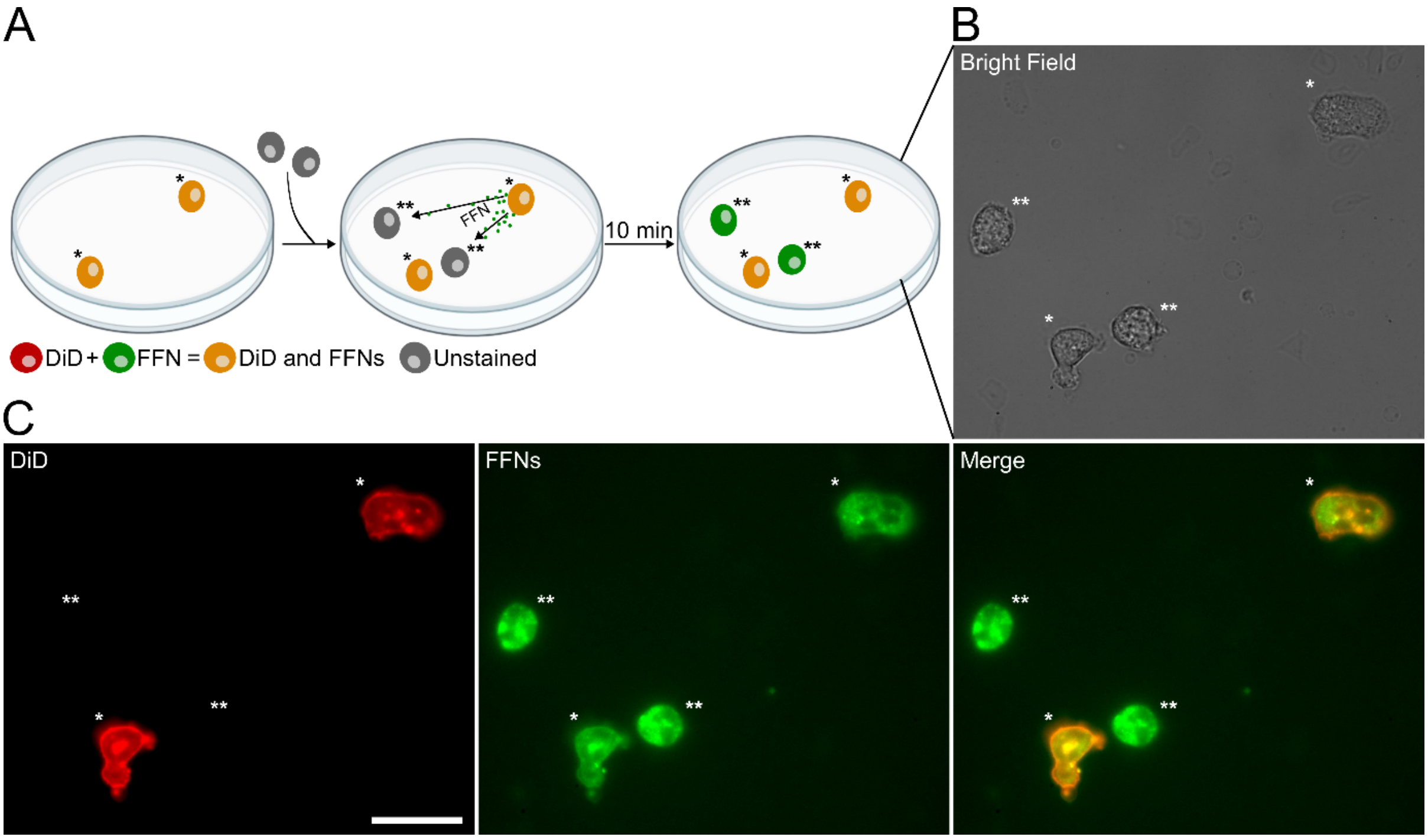

Fig. S16 *Intercellular transfer of FFNs between neutrophils:* ***(A)*** *Schematic illustration of the experimental setup: DiD-labeled neutrophils were loaded with FFN 511(orange) and washed before addition of freshly isolated, unstained (FFN-negative) neutrophils (grey).* ***(B)*** *Bright-field image of the co-culture at the experimental endpoint.* ***(C)*** *Endpoint immunofluorescence images showing the merged, DiD (red), and FFN (green) channels. FFNs from DiD-positive cells were transferred to previously unstained, DiD-negative neutrophils, which became FFN-positive after co-incubation, demonstrating intercellular FFN transfer. The DiD staining allows visualization of originally FFN-negative cells. Scale bar: 20 µm.*

Table S1: Antibodies used for immunofluorescence staining

**primary antibodies**	**supplier**	**concentration**
anti-β1-adrenergic receptor polyclonal antibody – rabbit IgG	Invitrogen, PA1-049	1:200
anti-β2-adrenergic receptor polyclonal antibody – rabbit IgG	Invitrogen, PA5-80323	1:1000
anti-catechol-O-methyltransferase polyclonal antibody – rabbit IgG	Novus Biologicals, NBP3-03726	1:100
anti-dopamine transporter polyclonal antibody – rabbit IgG	Proteintech, 22524-1-AP	1:200
anti-dopamine transporter antibody, culture supernatant – rat IgG2a κ	Sigma-Aldrich, MAB369	1:1000
anti-monoamine oxidase A monoclonal antibody – rabbit IgG	Abcam, ab126751	1:40
anti-monoamine oxidase B polyclonal antibody – rabbit IgG	Abcam, ab175136	1:100
anti-microtubule-associated protein 2 polyclonal antibody – chicken IgY	Novus Biologicals, NB300-213	1:2000
anti-tyrosine hydroxylase polyclonal antibody – rabbit antiserum	Synaptic Systems, 213102	1:500
anti-vesicular monoamine transporter 2 polyclonal antibody – rabbit IgG	Invitrogen, PA5-112713	1:200
anti-vesicular monoamine transporter 2 polyclonal antibody – rabbit IgG	Proteintech, 20873-1-AP	1:200
anti-vesicular monoamine transporter 2 antibody – rabbit IgG	Frontier Institute, AB2571857	1:2000
**isotypes**		
rabbit IgG monoclonal antibody	Abcam, ab172730	according to primary
rabbit IgG monoclonal antibody	Invitrogen, 08-6199	undiluted
**secondary antibodies**		
goat anti-rabbit IgG secondary antibody – Alexa Fluor™ 488	Invitrogen, A-11034	1:500
goat anti-mouse IgG secondary antibody – Alexa Fluor™ Plus 555	Invitrogen, A32727	1:1000

Table S2: Primer sequences for qPCR

gene	direction	sequence
ADRB1	forward	5‘-CCGGGAACAGGAACACAC-3‘
ADRB1	reverse	5‘-GAAAGCAAAAGGAAATATGTCTTGA-3‘
ADRB2	forward	5‘-TTGCCTCTTCCATCGTGTCC-3‘
ADRB2	reverse	5‘-CCACCTGGCTAAGGTTCTGG-3‘
B2M	forward	5‘-CCACTGAAAAAGATGAGTATGCCT-3‘
B2M	reverse	5‘-CCAATCCAAATGCGGCATCTTCA-3‘
COMT	forward	5‘-GGAGGCCATTGACACCTACTG-3‘
COMT	reverse	5‘-CGATCTTGCCTTTCTTGTCGC-3‘
MAOA	forward	5‘-AGGACTATCTGCTGCCAAAC-3‘
MAOA	reverse	5‘-AAGCTCCACCAACATCTACG-3‘
MAOB	forward	5‘-GCGGCATCTCAGGTATGGCA-3‘
MAOB	reverse	5‘-TCCAATCCTAGCTCCTTGGCT-3‘

Table S3: Thermal cycling profile for qPCR

step	temperature	duration	cycles
initial denaturation	95 °C	15 min	1
denaturation	95 °C	15 s	40
annealing	56 °C	20 s	
elongation	72 °C	20 s	
melting curve	60 – 95 °C, ΔT = 1 °C	10 s	1

Table S4: Parameters for simulation

**Parameter**	**Chosen Value**	**Source**
Simulation area:	256 µm x 256 µm	
Resolution (Pixel Size):	0.5 µm x 0.5 µm	
Simulated time	120 s	According to the experimental conditions
Placement of cell in simulation area	10 µm diameter, in the center of the	According to the experimental conditions
Density of SWCNT nanosensors	0.5 µm x 0.5 µm per sensor (1 per pixel)	based on *(56)*
Binding sites per nanosensor	30	based on *(57)*
k_{on}	V	
k_{off}	Varies between the different runs of the simulators	
DA diffusion coefficient	Varies between the different runs of the simulators	
Geometry of DA release	Varies between the different runs of the simulators	
Amount of DA molecules released	Varies between the different runs of the simulators	

Table S5: Variation of parameters for different runs of the diffusion simulation

	**k_{on}**	**k_{off}**	**Diffusion coefficient**	**Geometry of DA release**	**Amount of DA molecules released**
**Run 1**	10^6 $M^{-1}s^{-1}$ (based on *(56)*)	0.1 s^{-1} (based on*(56)*)	D=6.05*10^{-6} cm²/s (based on *(55)*)	Release from small vesicles at a random position on the edge of the cell, 30 release events every 2 s (own assumption, singular release events from different vesicles given e.g. in*(58)*)	30000 DA molecules per release event (as in *(58)*)
**Run 2**	10^4 $M^{-1}s^{-1}$ (own assumption of slower k_{on})	0.1 s^{-1} (based on *(56)*)	D=6.05*10^{-6} cm²/s (based on *(55)*)	Single release event, evenly distributed over whole cell area (own assumption)	10^6 DA molecules (own assumption of increased DA release)
**Run 3**	10^6 $M^{-1}s^{-1}$ (based on*(56)*)	0.01 s^{-1} (own assumption of slower k_{off})	D=6.05*10^{-6} cm²/s (based on*(55)*)	Release from small vesicles at a random position on the edge of the cell, 30 release events every 2 s (own assumption, singular release events from different vesicles given e.g. in*(58)*)	30000 DA molecules per release event (as in *(58)*)
**Run 4**	10^4 $M^{-1}s^{-1}$ (own assumption of slower k_{on})	0.01 s^{-1} (own assumption of slower k_{off})	D=6.05*10^{-6} cm²/s (based on*(55)*)	Single release event, evenly distributed over whole cell area (own assumption)	10^6 DA molecules (own assumption of increased DA release)
**Run 5**	10^4 $M^{-1}s^{-1}$ (own assumption of slower k_{on})	0.1 s^{-1} (based on*(56)*)	D=6.05*10^{-9} cm²/s (own assumption of drastically reduced diffusion speed due to space below cell)	Single release event, evenly distributed over whole cell area (own assumption)	10^6 DA molecules (own assumption of increased DA release)

Table S6: Genes used for the investigation of the transcriptional dynamics

Gene Symbol	Protein Name	UniProt ID	Ensembl ID
ADCY10	Adenylate Cyclase 10	Q96PN6	ENSG00000143199
ADCY2	Adenylate Cyclase 2	Q08462	ENSG00000078295
ADCY3	Adenylyl cyclase type 3	O60266	ENSG00000138031
ADCY4	Adenylate Cyclase 4	Q8NFM4	ENSG00000129467
ADCY5	Adenylate Cyclase 5	O95622	ENSG00000173175
ADCY6	Adenylate Cyclase 6	O43306	ENSG00000174233
ADCY7	Adenylate Cyclase 7	P51828	ENSG00000121281
ADCY8	Adenylate Cyclase 8	P40145	ENSG00000155897
ADCY9	Adenylate Cyclase 9	O60503	ENSG00000162104
ADRB1	Adrenoceptor Beta 1	P08588	ENSG00000043591
ADRB2	Adrenoceptor Beta 2	P07550	ENSG00000169252
AKT1	AKT Serine/Threonine Kinase 1	P31749	ENSG00000142208
AKT2	AKT Serine/Threonine Kinase 2	P31751	ENSG00000105221
AKT3	AKT Serine/Threonine Kinase 3	Q9Y243	ENSG00000117020
ALDH1B1	Aldehyde Dehydrogenase 1 Family Member B1	P30837	ENSG00000137124
ALDH2	Aldehyde Dehydrogenase 2 Family Member	P05091	ENSG00000111275
ALDH3A2	Aldehyde Dehydrogenase 3 Family Member A2	P51648	ENSG00000072210
ARRB2	Arrestin Beta 2	P32121	ENSG00000141480
BCL2A1	Bcl-2-related protein A1	Q16548	ENSG00000140379
C5AR1	C5a anaphylatoxin chemotactic receptor 1	P21730	ENSG00000197405
COMT	Catechol-O-Methyltransferase	P21964	ENSG00000093010
CREB1	CAMP Responsive Element Binding Protein 1	P16220	ENSG00000118260
CREB3	CAMP Responsive Element Binding Protein 3	O43889	ENSG00000107175
CREB5	CAMP Responsive Element Binding Protein 5	Q02930	ENSG00000146592
CSF3R	Granulocyte colony-stimulating factor receptor	Q99062	ENSG00000119535
CTNNB1	Catenin Beta 1	P35222	ENSG00000168036
CXCR1	C-X-C chemokine receptor type 1	P25024	ENSG00000163464
CXCR2	C-X-C chemokine receptor type 2	P25025	ENSG00000180871
DBH	Dopamine beta-hydroxylase	P09172	ENSG00000123454
DDC	Dopa Decarboxylase	P20711	ENSG00000132437
DRD1	Dopamine Receptor D1	P21728	ENSG00000184845
DRD2	Dopamine Receptor D2	P14416	ENSG00000149295
DRD3	Dopamine Receptor D3	P35462	ENSG00000151577
DRD4	Dopamine Receptor D4	P21917	ENSG00000069696
DRD5	Dopamine receptor D5	P21918	ENSG00000169676
ELANE	Neutrophil elastase	P08246	ENSG00000197561

Gene Symbol	Protein Name	UniProt ID	Ensembl ID
FCGR3A	Low affinity immunoglobulin gamma Fc region receptor III-A	P08637	ENSG00000203747
FPR1	Formyl peptide receptor 1	P21462	ENSG00000171051
FUT4	Alpha-(1,3)-fucosyltransferase 4	P22083	ENSG00000196371
GSK3A	Glycogen Synthase Kinase 3 Alpha	P49840	ENSG00000105723
GSK3B	Glycogen Synthase Kinase 3 Beta	P49841	ENSG00000082701
HTR1B	5-HT1Dβ-Rezeptor	P28222	ENSG00000135312
HTR1D	5-Hydroxytryptamine Receptor 1D	P28221	ENSG00000179546
HTR1E	5-Hydroxytryptamine (Serotonin) Receptor 1E, G Protein-Coupled	P28566	ENSG00000168830
HTR1F	5-Hydroxytryptamine Receptor 1F	P30939	ENSG00000179097
HTR2A	5-Hydroxytryptamine (Serotonin) Receptor 2A	P28223	ENSG00000102468
HTR2B	5-Hydroxytryptamine (Serotonin) Receptor 2B, G Protein-Coupled	P41595	ENSG00000135914
HTR2C	5-Hydroxytryptamine (Serotonin) Receptor 2C, G Protein-Coupled	P28335	ENSG00000147246
HTR4	5-Hydroxytryptamine Receptor 4	Q13639	ENSG00000164270
HTR5A	5-Hydroxytryptamine (serotonin) receptor 5A	P47898	ENSG00000157219
HTR6	5-Hydroxytryptamine (Serotonin) Receptor 6	P50406	ENSG00000158748
HTR7	5-Hydroxytryptamine Receptor 7	P34969	ENSG00000148680
IL1B	Interleukin-1 beta	P01584	ENSG00000125538
IL1R1	Interleukin-1 receptor type 1	P14778	ENSG00000115594
IL1R2	Interleukin-1 receptor type 2	P27930	ENSG00000115590
IL6	Interleukin-6	P05231	ENSG00000136244
ILK	Integrin Linked Kinase	Q13418	ENSG00000166333
IRAK3	Interleukin-1 receptor-associated kinase 3	Q9Y616	ENSG00000090376
ITGAM	Integrin alpha-M (CD11b)	P11215	ENSG00000169896
ITGB2	Integrin beta-2 (CD18)	P05107	ENSG00000160255
MAOA	Monoamine Oxidase A	P21397	ENSG00000189221
MAOB	Monoamine Oxidase B	P27338	ENSG00000069535
MMP9	Matrix metalloproteinase-9	P14780	ENSG00000100985
MPO	Myeloperoxidase	P05164	ENSG00000005381
NFKBIA	NF-kappa-B inhibitor alpha	P25963	ENSG00000100906
PAH	Phenylalanine Hydroxylase	P00439	ENSG00000171759
PLCB1	Phospholipase C Beta 1	Q9NQ66	ENSG00000182621
PLCB2	Phospholipase C Beta 2	Q00722	ENSG00000137841
PLCB3	Phospholipase C Beta 3	Q01970	ENSG00000149782
PLCB4	Phospholipase C Beta 4	Q15147	ENSG00000101333

Gene Symbol	Protein Name	UniProt ID	Ensembl ID
PLCD3	Phospholipase C Delta 3	Q8N3E9	ENSG00000161714
PLCD4	Phospholipase C Delta 4	Q9BRC7	ENSG00000115556
PLCE1	Phospholipase C epsilon 1	Q9P212	ENSG00000138193
PLCG1	Phospholipase C Gamma 1	P19174	ENSG00000124181
PLCG2	Phospholipase C Gamma 2	P16885	ENSG00000197943
PLCH2	phospholipase C eta 2	O75038	ENSG00000149527
PLCL1	Phospholipase C Like 1 (Inactive)	Q15111	ENSG00000115896
PLCL2	Phospholipase C Like 2	Q9UPR0	ENSG00000154822
PLCZ1	Phospholipase C Zeta 1	Q86YW0	ENSG00000139151
PNMT	Phenylethanolamine N-Methyltransferase	P11086	ENSG00000141744
PPP1CA	Protein Phosphatase 1 Catalytic Subunit Alpha	P62136	ENSG00000172531
PPP1CB	Protein Phosphatase 1 Catalytic Subunit Beta	P62140	ENSG00000213639
PPP1CC	Protein Phosphatase 1 Catalytic Subunit Gamma	P36873	ENSG00000186298
PPP1R1B	Protein Phosphatase 1 Regulatory Inhibitor Subunit 1B	Q9UD71	ENSG00000131771
PPP2CA	Protein Phosphatase 2 Catalytic Subunit Alpha	P67775	ENSG00000113575
PRKACA	Protein Kinase CAMP-Activated Catalytic Subunit Alpha	P17612	ENSG00000072062
PRKACB	Protein Kinase CAMP-Activated Catalytic Subunit Beta	P22694	ENSG00000142875
PRKACG	Protein Kinase CAMP-Activated Catalytic Subunit Gamma	P22612	ENSG00000165059
PRKAR1A	Protein Kinase CAMP-Dependent Type I Regulatory Subunit Alpha	P10644	ENSG00000108946
PRKAR2A	Protein Kinase CAMP-Dependent Type II Regulatory Subunit Alpha	P13861	ENSG00000114302
PRKAR2B	Protein Kinase CAMP-Dependent Type II Regulatory Subunit Beta	P31323	ENSG00000005249
PRKCA	Protein Kinase C Alpha	P17252	ENSG00000154229
PRKCB	Protein Kinase C Beta	P05771	ENSG00000166501
PRKCD	Protein Kinase C Delta	Q05655	ENSG00000163932
PRKCE	Protein Kinase C Epsilon	Q02156	ENSG00000171132
PRKCH	Protein Kinase C Eta	P24723	ENSG00000027075
PRKCI	Protein Kinase C Iota	P41743	ENSG00000163558
PRKCQ	Protein Kinase C Theta	Q04759	ENSG00000065675
PRKCZ	Protein Kinase C Zeta	Q05513	ENSG00000067606
PRTN3	Myeloblastin (Proteinase 3)	P24158	ENSG00000196415
PTGS2	prostaglandin-endoperoxide synthase 2	P35354	ENSG00000073756
S100A12	S100 Calcium Binding Protein A12	P80511	ENSG00000163221

Gene Symbol	Protein Name	UniProt ID	Ensembl ID
S100A8	S100 Calcium Binding Protein A8	P05109	ENSG00000143546
S100A9	S100 Calcium Binding Protein A9	P06702	ENSG00000163220
SLC6A3	Solute Carrier Family 6 Member 3	Q01959	ENSG00000142319
TH	Tyrosine Hydroxylase	P07101	ENSG00000180176
TLR4	Toll-like receptor 4	O00206	ENSG00000136869
TNF	Tumor necrosis factor	P01375	ENSG00000232810

Table S7: Genes of serotonin (5-HT) receptors used for the investigation of the transcriptional dynamics and their annotated functional mode on downstream signaling

Gene	Protein Name	Annotated functional mode on downstream signaling
HTR1B	5-Hydroxytryptamine (serotonin) Receptor 1B	inhibition of adenylate cyclase activity
HTR4	5-Hydroxytryptamine Receptor 4	activation of adenylate cyclase activity
HTR5A	5-Hydroxytryptamine (serotonin) Receptor 5A	inhibition of adenylate cyclase activity
HTR6	5-Hydroxytryptamine (Serotonin) Receptor 6	activation of adenylate cyclase activity
HTR1E	5-Hydroxytryptamine (Serotonin) Receptor 1E, G Protein-Coupled	inhibition of adenylate cyclase activity
HTR1D	5-Hydroxytryptamine Receptor 1D	inhibition of adenylate cyclase activity
HTR2C	5-Hydroxytryptamine (Serotonin) Receptor 2C, G Protein-Coupled	activation of phospholipase C-beta
HTR2C	5-Hydroxytryptamine (Serotonin) Receptor 2C, G Protein-Coupled	activation of phospholipase C-beta
HTR1A	5-Hydroxytryptamine Receptor 1A	inhibition of adenylate cyclase activity
HTR2B	5-Hydroxytryptamine (Serotonin) Receptor 2B, G Protein-Coupled	activation of phospholipase C-beta
HTR1F	5-Hydroxytryptamine Receptor 1F	inhibition of adenylate cyclase activity
HTR2A	5-Hydroxytryptamine (Serotonin) Receptor 2A	activation of phospholipase C-beta

Table S8: Genes used for the investigation of the transcriptional dynamics of inflammation scores.

Gene	Protein Name	UniProt ID	Ensembl ID
BCL2A1	Bcl-2-related protein A1	Q16548	ENSG00000140379
C5AR1	C5a anaphylatoxin chemotactic receptor 1	P21730	ENSG00000197405
CSF3R	Granulocyte colony-stimulating factor receptor	Q99062	ENSG00000119535
CXCR1	C-X-C chemokine receptor type 1	P25024	ENSG00000163464
CXCR2	C-X-C chemokine receptor type 2	P25025	ENSG00000180871
ELANE	Neutrophil elastase	P08246	ENSG00000197561
FCGR3A	Low affinity immunoglobulin gamma Fc region receptor III-A	P08637	ENSG00000203747
FPR1	Formyl peptide receptor 1	P21462	ENSG00000171049
FUT4	Alpha-(1,3)-fucosyltransferase 4	P22083	ENSG00000196371
IL1B	Interleukin-1 beta	P01584	ENSG00000125538
IL1R1	Interleukin-1 receptor type 1	P14778	ENSG00000115594
IL1R2	Interleukin-1 receptor type 2	P27930	ENSG00000115590
IL6	Interleukin-6	P05231	ENSG00000136244
IRAK3	Interleukin-1 receptor-associated kinase 3	Q9Y616	ENSG00000145362
ITGAM	Integrin alpha-M (CD11b)	P11215	ENSG00000169896
ITGB2	Integrin beta-2 (CD18)	P05107	ENSG00000160255
MMP9	Matrix metalloproteinase-9	P14780	ENSG00000100985
MPO	Myeloperoxidase	P05164	ENSG00000049239
NFKBIA	NF-kappa-B inhibitor alpha	P25963	ENSG00000100906
PRTN3	Myeloblastin (Proteinase 3)	P24158	ENSG00000196415
PTGS2	prostaglandin-endoperoxide synthase 2	P35354	ENSG00000073756
S100A12	S100 Calcium Binding Protein A12	P80511	ENSG00000163221

S100A8	S100 Calcium Binding Protein A8	P05109	ENSG00000143546
S100A9	S100 Calcium Binding Protein A9	P06702	ENSG00000163220
TLR4	Toll-like receptor 4	O00206	ENSG00000136869
TNF	Tumor necrosis factor	P01375	ENSG00000232810

Table S9: Number of donors and cells (in single cell experiments)

Figure		n [donors]	N [cells]
1B	DA	12	No single-cell data
	NE	6	
	E	9	
	HVA	6	
	VMA	6	
1D	5-HT	3	No single-cell data
	LPS	3	
1H	LatA + 5-HT	8	No single-cell data
	LatA + LPS	6	
	LatA	3	
1I	Control/5-HT	3	No single cell data
2F	Control	4	138
	LPS	4	49
	5-HT	3	35
	fMLP	4	51
2I	CA	2	30
	FFN102	2	30
3B	Control	3	No single-cell data
	DA	3	
	5-HT	6	
4C	5-HT	5	246
	Ketanserin	5	121
	CP809	3	96
4G	Control	No cells	No single-cell data, 18 ROIs
	Platelets	3	No single-cell data
	Platelets + Neutrophils	4	56
	Platelets + Neutrophils + Ketanserin	4	73
	Neutrophils	3	45

Figure		n [donors]	N [cells]
5B	Neutrophil lysate	5	No single-cell data
	NTC	3	
5C	ADRB1	5	No single-cell data
	ADRB2	3	
5D	DA 1 µM	3	No single-cell data
	DA 100 µM	6	
	NE 1 µM	3	
	NE 100 µM	6	
	E 1 µM	6	
	E 100 µM	9	
	5-HT 1 µM	5	
	5-HT 100 µM	5	
			No single-cell data
5F	Control	13	No single-cell data
	all others	8	
6	All	4	No single-cell data
S2	MAOA	6	No single-cell data
	MAOB	6	
	COMT	6	
S3	Supernatant	4	No single-cell data
	Lysate	4	
S4	Neutrophils/Inhibited Neutrophils	5	182/132
	HEK/Inhibited HEK	4	80/6
	HeLa/Inhibited HeLa	3	50/18
S6	Supernatant/ Lysate	4	No single-cell data
S7	5-HT	1	10
	Control	1	3

**Figure**		**n [donors]**	**N [cells]**
S9	1	5	15
	2	5	27
	3	5	150
	4	5	16
	5	5	25
S12C	Ionomycin	1	11
S14	Control	14	No single cell data
	all others	8	
S15	all	4	No single-cell data
